# Host 3’ flap endonuclease Mus81 plays a critical role in trimming the terminal redundancy of hepatitis B virus relaxed circular DNA during covalently closed circular DNA formation

**DOI:** 10.1371/journal.ppat.1012918

**Published:** 2025-02-06

**Authors:** Hu Zhang, Quanxin Long, Yuanjie Liu, Alexander L. Marchetti, Cheng-Der Liu, Ning Sun, Haitao Guo

**Affiliations:** 1 Department of Microbiology and Molecular Genetics; Cancer Virology Program, UPMC Hillman Cancer Center, University of Pittsburgh School of Medicine, Pittsburgh, Pennsylvania, United States of America; 2 Department of Microbiology and Immunology, Indiana University School of Medicine, Indianapolis, Indiana, United States of America; Pennsylvania State University College of Medicine: Penn State College of Medicine, UNITED STATES OF AMERICA

## Abstract

Hepatitis B virus (HBV) relaxed circular DNA (rcDNA) possesses an 8–9 nucleotide-long terminal redundancy (TR, or *r*) on the negative (-) strand DNA derived from the reverse transcription of viral pregenomic RNA (pgRNA). It remains unclear whether the TR forms a 5’ or 3’ flap structure on HBV rcDNA and which TR copy is removed during covalently closed circular DNA (cccDNA) formation. To address these questions, a mutant HBV cell line HepDES-C1822G was established with a C1822G mutation in the pgRNA coding sequence, altering the sequence of 3’ TR of (-) strand DNA while the 5’ TR remained wild type (wt). The production of HBV rcDNA and cccDNA in HepDES-C1822G cells was comparable to wt levels. Next-generation sequencing (NGS) analysis revealed that the positive (+) strand DNA of rcDNA and both strands of cccDNA predominantly carried the wt nt1822 residue, indicating that the 5’ TR of (-) strand DNA serves as the template during rcDNA replication, forming a duplex with the (+) strand DNA, while the 3’ TR forms a flap-like structure, which is subsequently removed during cccDNA formation. In a survey of known cellular flap endonucleases using a loss-of-function study, we found that the 3’ flap endonuclease Mus81 plays a critical role in cccDNA formation in wild-type HBV replicating cells, alongside the 5’ flap endonuclease FEN1. Additionally, we have mapped the potential Mus81 and FEN1 cleavage sites within the TR of nuclear DP-rcDNA by RACE-NGS analyses. The overlapping function between Mus81 and FEN1 in cccDNA formation indicates that the putative 5’ and 3’ flap formed by TR are dynamically interchangeable on rcDNA precursor. These findings shed light on HBV rcDNA structure and cccDNA formation mechanisms, contributing to our understanding of HBV replication cycle.

## Author summary

HBV cccDNA is the persistent form of viral genome and the basis for viral rebound after the cessation of therapy, thus, understanding the biosynthesis of cccDNA is crucial for the development of novel antiviral therapeutics to cure chronic HBV infection. We report herein that the 5’ copy of HBV negative strand DNA terminal redundancy (TR, or *r*) is selected for the asymmetric positive strand DNA synthesis, while the 3’ copy of TR is preferentially removed during rcDNA-to-cccDNA conversion. Consistent with that, we found that Mus81, a cellular 3’ flap endonuclease, plays a critical role in cccDNA biosynthesis. Furthermore, the observed overlapping function of Mus81 and 5’ flap endonuclease FEN1 in cccDNA formation indicates a dynamic interchange between TR-derived 5’ and 3’ flap on rcDNA in this process. These findings enhance our understanding of HBV replication, offering new insights for the development of innovative antiviral therapeutics targeting cccDNA.

## Introduction

Human hepatitis B is a potentially life-threatening liver infection caused by hepatitis B virus (HBV), which can cause chronic infection and puts people at high-risk occurrence of cirrhosis and deadly hepatocellular carcinoma [1]. 296 million people were living with chronic hepatitis B in 2019, with an estimated 820,000 deaths and 1.5 million new infections each year [2]. While hepatitis B can be prevented through vaccination, individuals who are already chronically infected with HBV are difficult to cure [3].

HBV is the prototype member of the *Hepadnaviridae* family and a para-retrovirus that replicates its DNA genome *via* reverse transcription of the pregenomic RNA (pgRNA) encoded by viral covalently closed circular DNA (cccDNA), which is the persistent form of HBV genome and the authentic transcription template in HBV-infected hepatocyte [4]. Currently approved HBV therapies with nucleos(t)ide reverse transcriptase inhibitors (NUCs) and/or interferon-alpha (IFNα) can effectively reduce virus load and slow down disease progression but fail to eliminate the viral infection, specifically the cccDNA, in the majority of treated patients [5]. Although various compounds targeting different steps of HBV life cycle have been developed and some have advanced to clinical trials, but similar to NUCs and IFNα, the lack of direct and potent inhibitory effect of those drugs on cccDNA leaves HBV cure still a challenge [6,7]. HBV cccDNA exists in the nucleus of infected hepatocytes as a chromatin-like minichromosome, which functions to transcribe viral RNAs and supports viral replication [8,9]. The persistence of a functional cccDNA pool is responsible for treatment failure and viral rebound after the treatment withdrawal [10–12]. Therefore, understanding the mechanisms underlying cccDNA biosynthesis, maintenance and transcription regulation is essential for development of novel antiviral therapeutics to cure chronic hepatitis B [9,12–16].

**Fig 1:**
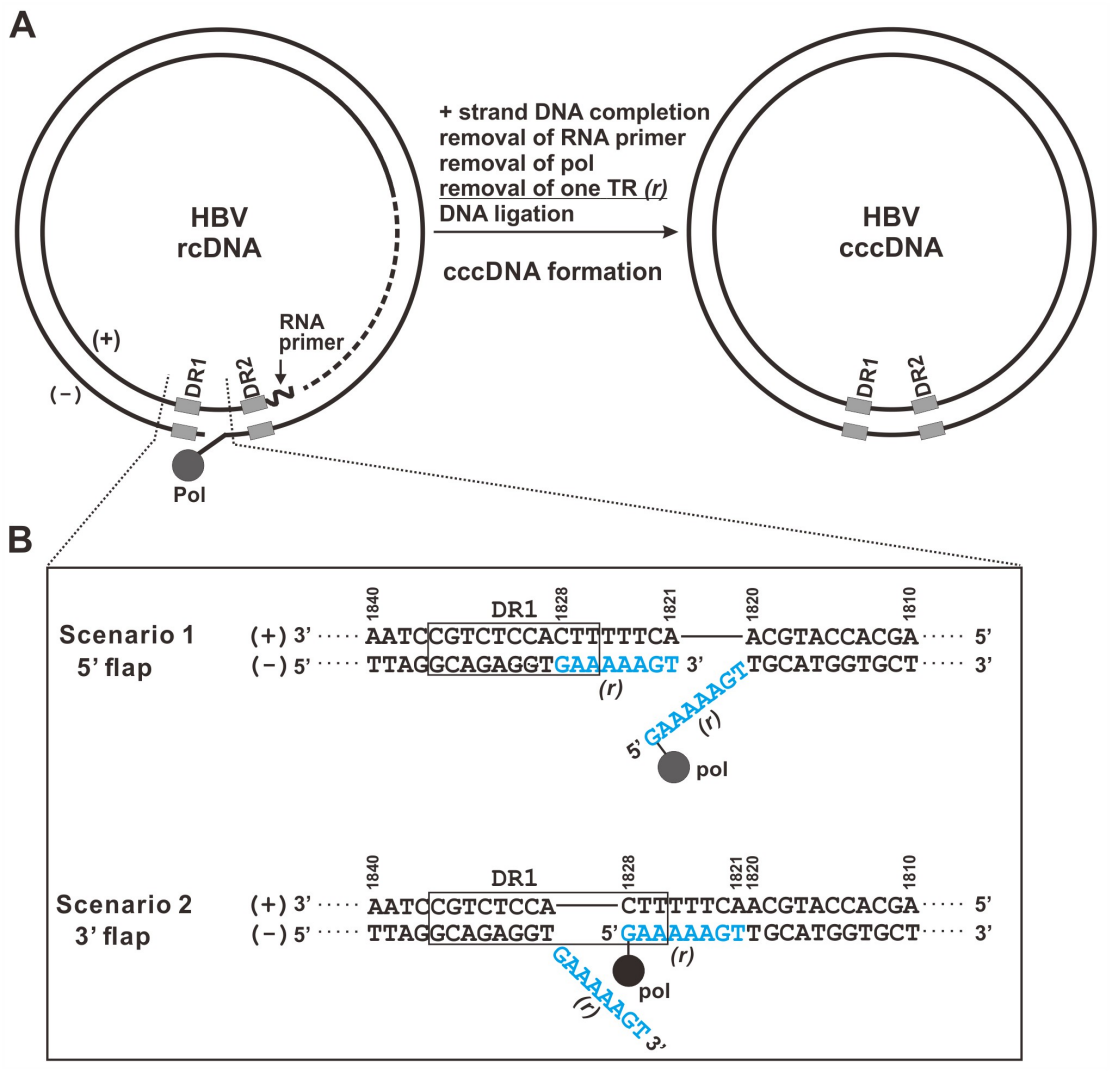
Schematic illustrations of HBV rcDNA, terminal redundancy (TR or *r*), and cccDNA. (A) The positive (+) strand and negative (-) strand of rcDNA are labeled. The broken line indicates varying lengths of (+) strand DNA. The RNA primer at the 5’ end of (+) strand is depicted with a curved line. Viral polymerase (Pol) covalently attached to the 5’ end of (-) strand is indicated by a solid dot. DR1 and DR2 motifs are shown as gray rectangles. The mandatory molecular reactions required for converting rcDNA into cccDNA are listed. (B) A sequence fragment (nt 1810–1840) flanking the TR region (nt 1821–1828, shown in blue letters) of the genotype D HBV genome (GenBank Accession No.: U95551.1) is displayed. Depending on the base pairing between (+) strand DNA and 3’ or 5’ TR, a DNA flap stucture forms on the 5’ or 3’ end of (-) strand DNA of rcDNA, respectively.

Unlike host chromosomal DNA and other viruses with double-stranded circular DNA genomes such as papillomaviruses and polyomaviruses [17,18], the authentic HBV cccDNA does not undergo semi-conservative replication but is only converted from viral relaxed circular DNA (rcDNA) during *de novo* infection and/or *via* the intracellular amplification pathway (also known as (aka) rcDNA recycling pathway) [4,19,20]. The rcDNA is a 3.2 kb partially double-stranded open circular viral DNA genome within the mature capsid and virion particles, and it is the major final product from reverse transcribing the pgRNA template catalyzed by viral polymerase (Pol) [21–23] (Fig 1). To date, the molecular mechanism by which rcDNA is converted into cccDNA is still not fully understood. Comparing the major differences between rcDNA and cccDNA (Fig 1A), a series of biological reactions are required to correct the molecular peculiarities on rcDNA termini during cccDNA formation, including 1) completion of positive (+) strand DNA synthesis; 2) removal of the 5’ end capped RNA primer on (+) strand DNA; 3) removal of viral Pol covalently attached to the 5’ end of negative (-) strand DNA; 4) removal of one copy of the terminal redundancy (TR, or *r*) on (-) strand DNA; 5) ligation of both strands to generate the *bona fide* cccDNA [13,14]. Among them, the 8–9 nucleotide (nt)-long TR on (-) strand DNA is a duplication of nt 1820/1821 (transcription initiation sites) to nt 1828 (reverse transcription initiation site) reversed transcribed from the pgRNA template, and the 5’ TR is covalently linked to Pol as a result of protein-primed DNA synthesis [19,21]. However, the (+) strand DNA only contains one complementary copy of TR as its internal sequence, indicating a template selection of TR during (+) strand DNA synthesis. Since only one copy of TR will form duplex with (+) strand DNA at the cohesive end region of rcDNA, it remains unclear whether TR forms a 5’ or 3’ flap-like structure on rcDNA and which copy of TR is discarded during cccDNA formation (Fig 1B). A previous study introduced mutations into the 5’ terminus of duck hepatitis B virus (DHBV) rcDNA (-) strand and the sequence analysis of mutant virus-derived rcDNA and cccDNA revealed that in a high frequency (~ 85%), the (+) strand DNA synthesis used the mutated 5’ TR as template; and the mutant 5’ TR sequence was largely replaced by wild type (wt) 3’ TR sequence in cccDNA, indicating a preferential removal of mutant 5’ TR of rcDNA during DHBV cccDNA formation [24]. However, given the different viral DNA sequences and host cell background between DHBV and HBV, whether the above findings from DHBV mutagenesis study also apply to HBV remains unexplored.

It is generally acknowledged that HBV relies on host DNA repair machinery to convert rcDNA into cccDNA and recent studies in this direction have identified a handful of cellular DNA repair factors and viral DNA intermediates involved in cccDNA biosynthesis (reviewed in [12,19,20]). Among them, a deproteinated rcDNA (DP-rcDNA, or protein-free rcDNA) species, which is devoid of the covalently attached Pol at 5’ end of (-) strand DNA, has been previously identified and suggested as an intermediate during cccDNA formation [25–30]. The in-depth analyses of cytoplasmic DP-rcDNA termini revealed that both copies of TR sequence remain intact on rcDNA, indicating that the removal of one copy of TR occurs after the import of DP-rcDNA into nucleus [31]. Furthermore, a closed minus-strand rcDNA (CM-rcDNA), which possesses a covalently closed (-) strand containing single copy of TR but an unligated (+) strand, has been discovered as a minor species of DP-rcDNA population *via* exonuclease digestion, indicating that the TR excision and (-) strand ligation may take place prior to the completion of (+) strand DNA repair during cccDNA formation [32,33]. Despite the unavailability of an exact topological information of the putative terminal flap structure on HBV rcDNA, previous studies, likely inspired by the aforementioned DHBV study [24], have demonstrated that the 5’ flap structure-specific endonuclease 1 (FEN1) plays a role in HBV cccDNA production in cell-based phenotypic assays and in *in vitro* biochemical assays using synthetic rcDNA substrates with a preset 5’ flap structure [34,35]. However, chemical inhibition or siRNA knockdown (~80% efficiency) of FEN1 only partially reduced cccDNA formation (≤50%) in HBV cell culture models [34], inferring that additional viral and cellular factors may be involved in determining TR excision during cccDNA formation.

In the current study, we set out to investigate the template selection of TR on HBV (-) strand DNA during (+) strand DNA synthesis and cccDNA formation. We reveal that the 5’ TR of HBV (-) DNA is predominantly chosen for asymmetric (+) strand DNA synthesis. Interestingly, during the conversion of rcDNA to cccDNA, the 3’ copy of TR is selectively eliminated. In line with this, we demonstrate that Mus81, a cellular 3’ flap endonuclease, plays a critical role in cccDNA biosynthesis in addition to FEN1. Additionally, we have mapped the potential FEN1 and Mus81 cleavage sites within TR of the nuclear DP-rcDNA. Altogether, our findings suggest an overlapping function between Mus81 and FEN1 in cccDNA formation, indicating a plausible dynamic exchange between TR-derived 5’ and 3’ flaps on rcDNA during this process. These discoveries deepen our understanding of HBV replication mechanisms and provide valuable insights for the development of novel antiviral therapies targeting cccDNA.

## Results

### Producing rcDNA with a point mutation in 3’ TR

**Fig 2:**
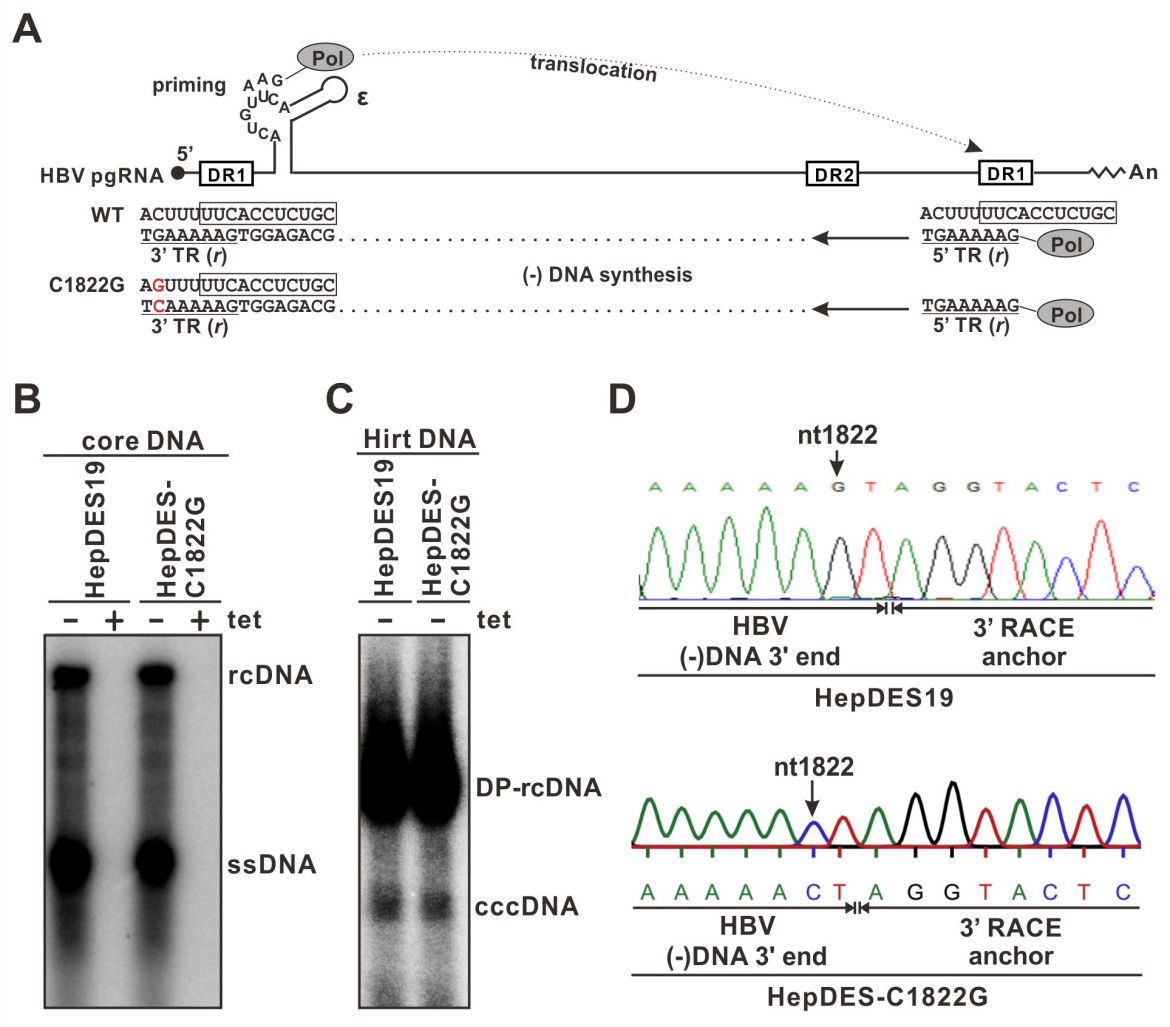
Establishment of HepDES-C1822G cell line. (A) Mutagenesis strategy for introducing a point mutation G1822C into the 3’ TR of rcDNA. HBV pgRNA is schematically illustrated, with the DR1/DR2 motifs and epsilon (ε) stem-loop structure being indicated. The C1822G mutation (in red) is introduced into the 5’ end of pgRNA coding sequence, which will be reverse transcribed into 1822C (in red) in the 3’ TR of (-) strand DNA, while the 5’ TR maintains a wild type 1822G. (B-C) HepDES19 cell line and the established HepDES-C1822G cell line were uninduced (+ tet) or induced (- tet) for 14 days. The tet-inducible (tet-off) HBV core DNA replication (B) and cccDNA formation (C) were analyzed by Southern blot. (D) HBV core DNA from the induced HepDES19 and HepDES-C1822G cells were subjected to (-) strand DNA 3’ RACE and Sanger sequencing. The RACE anchor-HBV 3’ TR junction sequences are labeled on the sequence chromatograms. Arrows indicate the wt 1822G and mutant 1822C in the 3’ TR of HBV (-) strand DNA from induced HepDES19 and HepDES-C1822G cells, respectively.

To investigate the TR selection of HBV (-) strand DNA during rcDNA and cccDNA biosynthesis, we introduced a point mutation into the 3’ TR (Fig 2A). The rationale for mutating 3’ TR instead of the 5’ one is that the latter requires mutating the priming site in 5’ epsilon or the 5–6 nt immediately upstream of 3’ DR1 on pgRNA, which contains important *cis* element for the initiation of viral reverse transcription [21]. In line with this, a previous study mutating the 5’ TR of DHBV genome by altering the priming site in 5’ epsilon resulted in certain level of aberrant priming and reduction of DHBV DNA replication [24]. Based on the known molecular mechanism of HBV rcDNA biosynthesis (Fig 2A), we designed a point mutation C1822G downstream of pgRNA transcription starting site (A1820/1821). During HBV DNA replication, the point mutation C1822G at the 5’ end of pgRNA will be reverse transcribed into G1822C in the 3’ TR of (-) DNA, while the 5’ TR maintains the wt G1822 (Fig 2A). Accordingly, we introduced the 5’ C1822G mutation into plasmid pTREHBVDES and established a HepG2-based, tetracycline (tet)-inducible HBV stable cell line HepDES-C1822G as described in Materials and Methods. Compared to the prototype HBV cell line HepDES19, the HepDES-C1822G line exhibited similar levels of cytoplasmic core DNA replication and nuclear cccDNA production upon induction (tet-) (Fig 2B and 2C). The 3’ RACE and sequencing of (-) strand DNA confirmed the G1822C mutation in the 3’ TR of rcDNA produced in HepDES-C1822G cells (Fig 2D).

### Template selection of (-) strand DNA TR during (+) strand DNA synthesis and cccDNA formation

**Fig 3:**
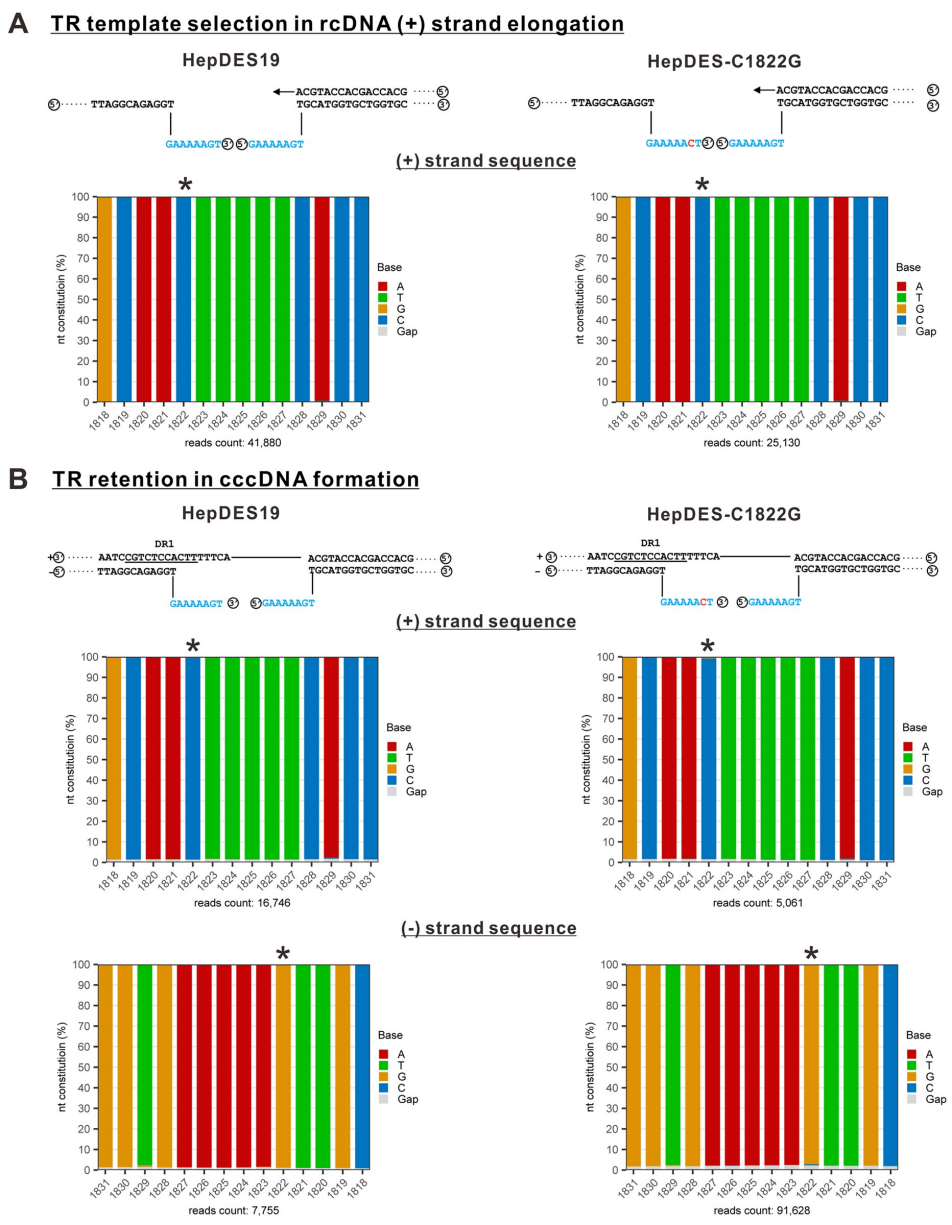
NGS analyses of HBV TR sequences in HepDES19 and HepDES-C1822G cells. (A) Selection of TR template for (+) strand DNA synthesis. The cytoplasmic HBV core DNA was extracted from the induced HepDES19 and HepDES-C1822G cells and subjected to (+) strand-specific PCR amplification of a 458-bp fragment containing the complementary sequence of TR region (S1A Fig). The PCR amplicon was sequenced by NGS and the nucleotide constitution at each position within nt 1812–1825 was plotted. The column of nt 1822 nucleotide constitution is marked with an asterisk. (B) Selection of TR for retention on cccDNA. Total Hirt DNA extracted from the induced HepDES19 and HepDES-C1822G cells were subjected to heat denaturation and PSAD treatment to remove DP-rcDNA, followed by PCR amplification of a 211-bp cccDNA fragment flanking the TR region (S1B Fig). NGS of the PCR amplicon was performed, and the nucleotide constitution at each position within nt 1812–1825 of both strands was plotted. Asterisks mark the columns representing nucleotide constitution at nt 1822 on both (+) and (-) strands of cccDNA.

The completion of HBV (-) strand DNA synthesis is accompanied by the degradation of pgRNA template catalyzed by Pol’s RNaseH activity, then the 5’ DR1-containing RNA remnant serves as primer to initiate rcDNA (+) strand synthesis after annealing with DR2, and once the (+) strand elongates to the TR region of (-) strand DNA, it needs to select one copy of TR (5’ or 3’ TR) as the template (Fig 1). To determine which TR is selected as the template for (+) strand DNA elongation during rcDNA synthesis, we developed a strategy to specifically amplify a (+) strand region spanning TR on rcDNA but not the double-stranded linear (dls) DNA (S1A Fig, upper panels). The viral dslDNA is synthesized as a byproduct through *in situ* priming of (+) DNA from the 3’ DR1 of (-) strand DNA without template switching to 5’ DR2 and, therefore, no TR selection is involved [19,23]. The amplification products were verified by agarose gel electrophoresis (S1A Fig, bottom panel), and then purified and subjected to next-generation sequencing (NGS). Among the clean sequencing reads, nt 1822 of rcDNA (+) strand was predominantly identified as a C in both wt (99.58%) and C1822G mutant (99.53%) amplicons (Fig 3A), indicating a preferential selection of 5’ TR for (+) strand elongation during rcDNA synthesis.

To determine which TR is removed from the (-) strand DNA of rcDNA during cccDNA formation, we designed a primer pair flanking the TR region (S1B Fig, upper panel). To prevent the unwanted PCR amplification from rcDNA, the HBV Hirt DNA samples were heated at 85°C to denature the DP-rcDNA into single-stranded (ss) DNA, followed by Plasmid-safe ATP-dependent DNase (PSAD) treatment to remove ssDNA and other non-cccDNA species [36]. The cccDNA PCR products were verified by agarose gel electrophoresis (S1B Fig, bottom panel). Both (+) and (-) strands of the cccDNA PCR product were sequenced by NGS. Among the (+) strand clean sequencing reads, the position 1822 was predominantly C in cccDNA from both HepDES19 (98.61%) and HepDES-C1822G (98.60%) cells (Fig 3B, middle panel), which was consistent with the above rcDNA (+) strand NGS results (Fig 3A). In terms of the cccDNA (-) strand NGS results, nt 1822 was predominantly a G in samples from both HepDES19 (98.57%) and HepDES-C1822G (97.92%) cells (Fig 3B, bottom panel). These findings align with the NGS results of both the cccDNA (+) strand and rcDNA (+) strand, reinforcing the observation that the 5’ TR sequence is predominantly favored as the template for rcDNA (+) strand synthesis and further retained within cccDNA, while the 3’ TR is preferentially removed during cccDNA formation, likely by a 3’ flap endonuclease.

### 3’ flap endonuclease XPF is dispensable for HBV cccDNA formation

**Fig 4:**
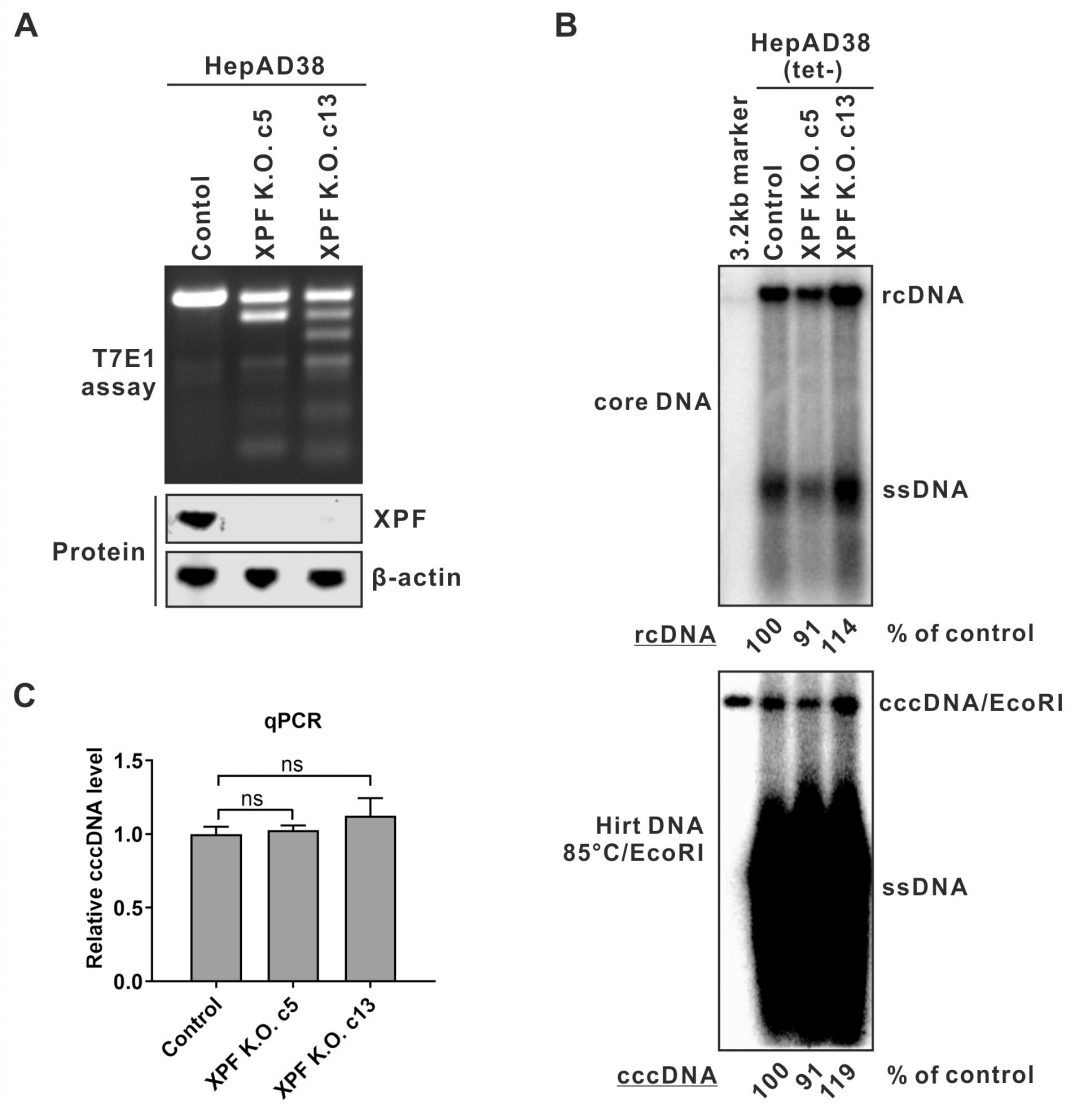
XPF knockout has no influence on HBV cccDNA production. (A) Verification of XPF knockout. The XPF gene editing and protein expression in HepAD38 control K.O. cells and XPF K.O. cell clone 5 (c5) and 13 (c13) were analyzed by T7E1 assay and Western blot, respectively. β-actin served as Western blot loading control. (B) HepAD38 control and XPF K.O. cells were cultured in tet-free medium for 14 days. HBV cytoplasmic core DNA and total Hirt DNA were extracted and detected by Southern blot. A 3.2 kb HBV DNA served as size marker. Prior to gel loading, the Hirt DNA samples were heat denatured to convert DP-rcDNA into ssDNA, followed by EcoRI digestion to linearize cccDNA into the unit-length dslDNA. The band intensities of cytoplasmic rcDNA and nuclear cccDNA were quantified and their relative levels were expressed as percentage (%) of control samples. (C) A portion of the Hirt DNA samples were heat denatured and digested by PSAD to remove DP-rcDNA. The remaining cccDNA was quantified by qPCR and normalized by total Hirt DNA qPCR. The relative cccDNA levels were plotted (control was set to 1) (mean ± SD, n = 3; ns: not significant).

Most eukaryotes possess two types of 3’ flap endonucleases, present as distinct heterodimeric complexes, namely the xeroderma pigmentosum group F-complementing protein and excision repair cross-complementing group 1 protein complex (XPF-ERCC1, simplified as XPF thereafter), and the MMS and UV-sensitive protein 81 and essential meiotic endonuclease 1 complex (Mus81-Eme1, simplified as Mus81 thereafter); each complex contains one catalytic and one non-catalytic subunit, and both exhibit endonuclease activity toward a variety of 3’ flap or fork DNA structures. [37]. The structure-specific endonuclease XPF and Mus81 play overlapping but essential roles in DNA repair by homologous recombination [38]. In our study, we first knocked out the XPF gene in HepAD38 cells by CRISPR and obtained two candidate XPF K.O. cell clones (c5 and c13), followed by K.O. validation. The T7 endonuclease I (T7E1) mismatch detection assay was employed to evaluate the CRISPR efficiency at the XPF gRNA target site, and the results showed that the cleavage of PCR products of the CRISPR-targeting gene fragment in XPF K.O. cells, indicating the presence of mutations in the XPF gene loci (Fig 4A, upper panel). Additionally, Western blotting demonstrated that XPF expression was eliminated in XPF K.O. cells (Fig 4A, lower panel). The indel mutation sequencing results further confirmed the successful knockout of XPF gene in HepAD38 XPF K.O. cells (S2 Fig).

We then examined the effect of XPF knockout on HBV replication and cccDNA formation by performing Southern blot and qPCR analyses. In order to highlight the cccDNA signal for Southern blot detection, the Hirt DNA sample underwent heat denaturation and subsequent digestion by EcoRI (S3A Fig). This procedure shifted down DP-rcDNA (major) and DP-dslDNA (minor) to the ssDNA position and shifted up cccDNA to the dslDNA position on Southern blot (S3B Fig, left half) [39]. Furthermore, the cytoplasmic core DNA received the same treatment did not show any distinguished band at the dslDNA position or above ssDNA (S3B Fig, right half), which ruled out a possibility that the two strands of denatured rcDNA (and DP-rcDNA) reanneal back into dslDNA during or after the treatment procedure.

The Southern blot and qPCR results showed that the levels of HBV rcDNA replicative intermediate and cccDNA product in XPF K.O. cells were comparable to those in control cells, and the levels of cccDNA were proportional to rcDNA levels in both control and K.O. cells (Fig 4B and 4C), demonstrating that the loss of XPF does not obviously affect HBV cccDNA formation, and therefore, XPF is unlikely responsible for rcDNA 3’ TR removal during cccDNA formation.

### Mus81 plays a critical role in HBV cccDNA intracellular amplification

**Fig 5:**
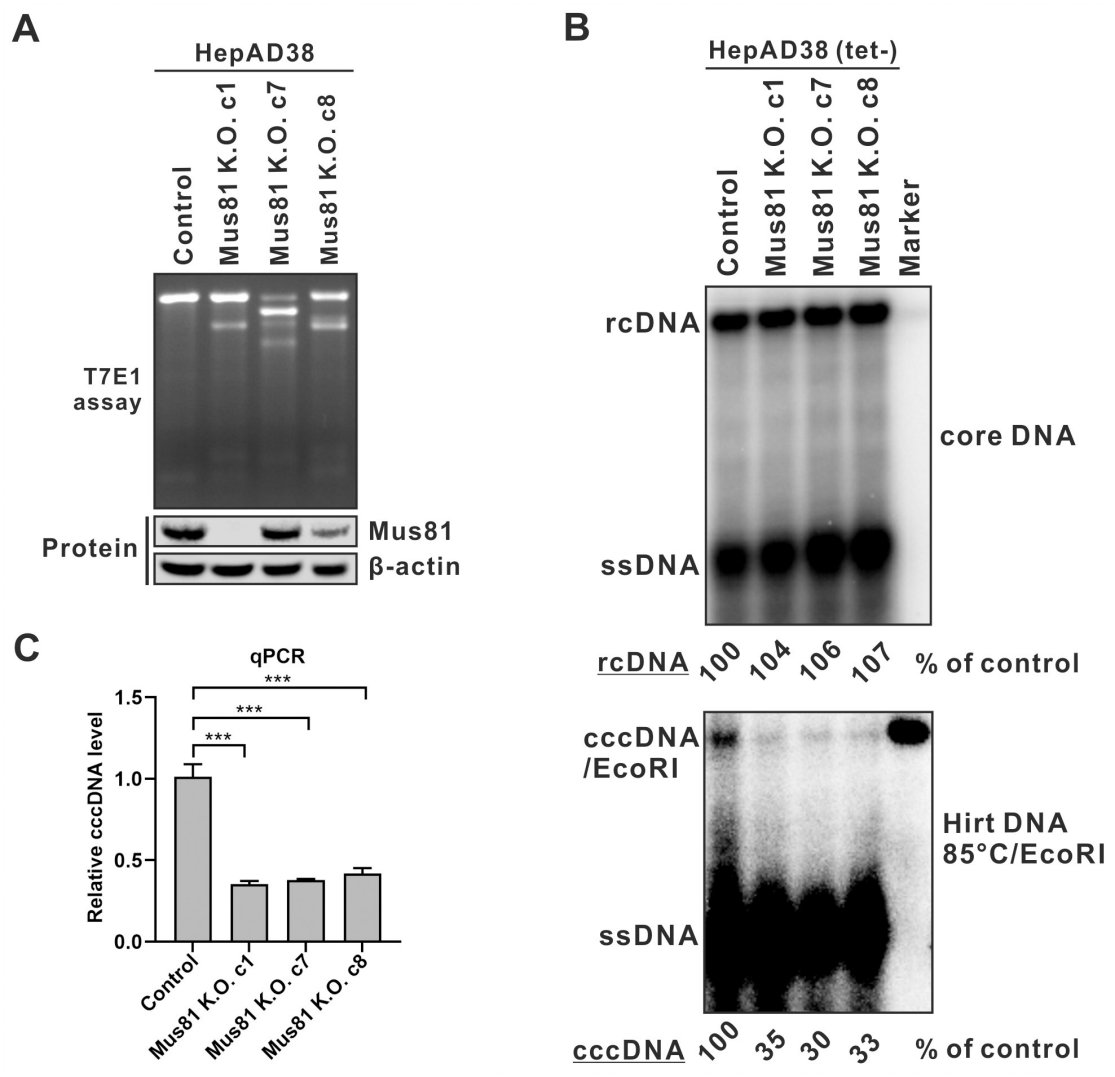
Mus81 knockout reduces HBV cccDNA production. (A) Verification of Mus81 knockout. Mus81 gene editing and protein expression in HepAD38 control K.O. cells and Mus81 K.O. clone 1 (c1), clone 7 (c7), and clone 8 (c8) were verified by T7E1 assay and Western blot, respectively. (B-C) HepAD38 control and Mus81 K.O. cells were induced in tet-free medium for 14 days, followed by (B) HBV core DNA and cccDNA Southern blot analyses and (C) cccDNA qPCR assay (mean ± SD, n = 3; ***p<0.001).

We obtained three Mus81 K.O. clones (c1, c7, and c8) in the background of HepAD38 cells as determined by T7E1 assay (Fig 5A, upper panel). However, the Western blot assay showed that the Mus81 expression was eliminated only in clone c1, whereas the Mus81 protein level was not affected in clone c7 clone, and clone c8 only exhibited a partial knockdown of Mus81 expression (Fig 5A, lower panel). By sequencing the CRISPR targeting loci, we found that the mutation in Mus81 K.O. c1 caused an ORF frameshift, resulting in a premature stop codon; however, the deletion mutants in c7 and c8 clones did not cause a frameshift, resulting in the expression of Mus81 protein with small internal deletions (S4 Fig).

Nonetheless, since the sgRNA was designed to target the coding sequence of Mus81 incision activity domain, the deletions in clones c7 and c8 likely disabled the enzyme activity of the expressed mutant Mus81 [40]. In this regard, Southern blot assay demonstrated that, while the HBV core DNA replication was not affected in all three HepAD38 Mus81 K.O. clones compared to the control K.O. cells, the cccDNA production was significantly decreased in Mus81 K.O. cells with a reduction of approximately 70% relative to the levels of rcDNA precursor (Fig 5B). The cccDNA qPCR results were largely consistent with the Southern blot date (Fig 5C). These findings suggest a crucial role for Mus81 in cccDNA formation *via* intracellular rcDNA recycling, and the presence of residual cccDNA in Mus81 knockout cells implies a potential compensatory or overlapping cellular function for Mus81.

### Mus81 plays a significant role in cccDNA formation during HBV infection *in vitro*

**Fig 6:**
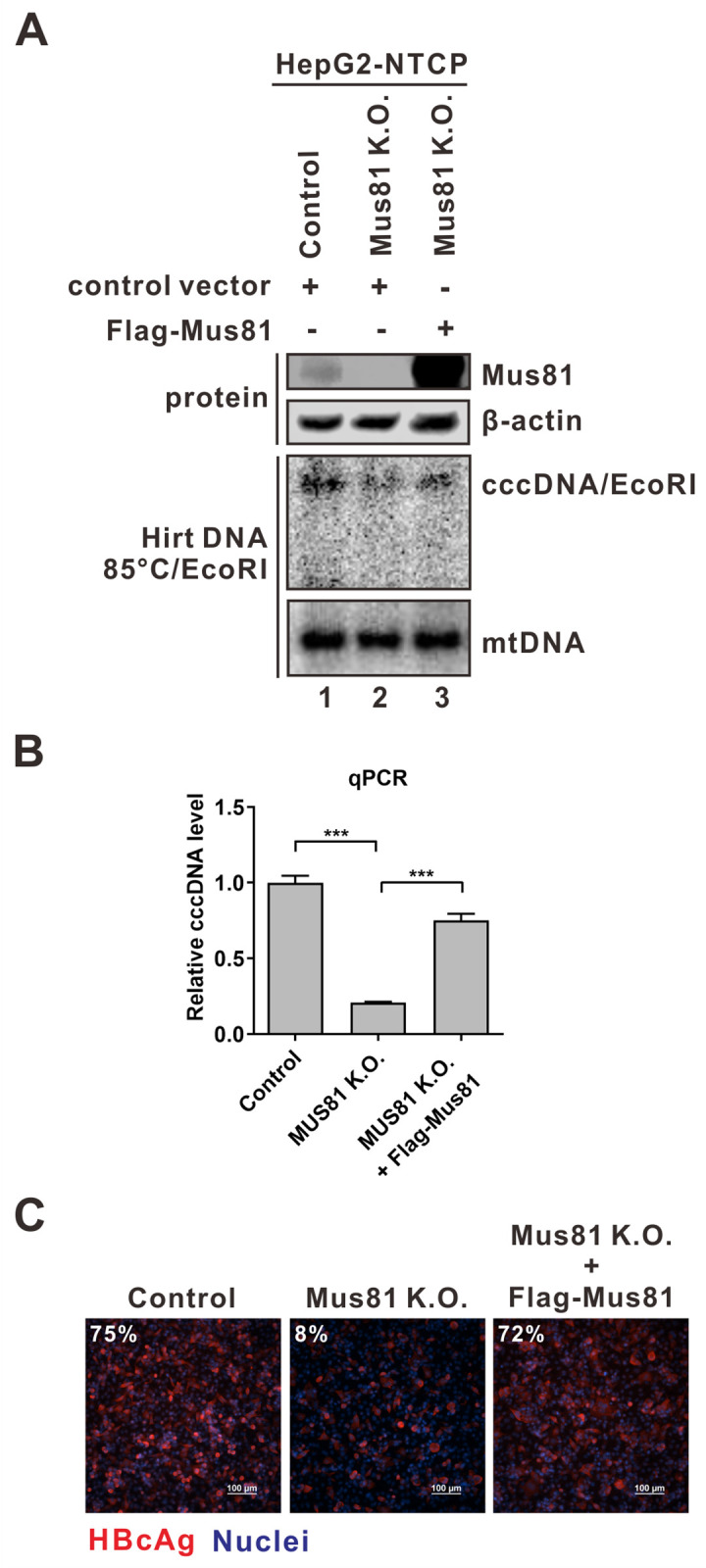
Mus81 knockout inhibits *de novo* cccDNA formation in HBV infection system. HepG2-NTCP control and Mus81 K.O. cells were transfected with control vector or plasmid expressing Flag-tagged Mus81. Two days post-transfection, the cells were infected with HBV at MOI of 200 for 5 days and subjected to the following analyses. (A) Protein expression of Mus81 was analyzed by Western blot with β-actin serving as loading control, and cccDNA was detected by Southern blot with mitochondrial DNA (mtDNA) serving as loading control. (B) cccDNA qPCR (mean ± SD, n = 3; ***p<0.001). (C) HBc immunofluorescence assay. Cell nuclei were counterstained with DAPI. The percentage of HBc-positive cells is indicated. Scale bar: 100 μm.

Next, we CRISPRed out Mus81 in HepG2-NTCP cells and obtained clone c7 with complete knockout of Mus81 expression, as validated by T7E1, Western blot, and Sanger sequencing assays (Figs 6A and S5). The obtained HepG2-NTCP Mus81 K.O. cells and control K.O. cells were then infected by HBV. As shown in Fig 6, the loss of Mus81 in HepG2-NTCP cells led to a significant reduction of cccDNA production (panel A-B) and HBcAg expression (panel C) compared to the control K.O. cells. Furthermore, transcomplementation of Mus81 expression by transfection rescued HBV infection in HepG2-NTCP Mus81 K.O. cells, which ruled out a possible off-target effect of Mus81 CRISPR approach (Fig 6A–6C).

To eliminate another possibility that knocking out Mus81 might have affected HBV entry, a step preceding cccDNA formation during *de novo* infection, we conducted hepatitis delta virus (HDV) infection in HepG2-NTCP Mus81 K.O. cells. HDV is a satellite RNA virus of HBV, which is coated by HBV envelope proteins and, thus, also utilizes NTCP as a receptor for entry but distinguishes itself through a unique post-entry replication mechanism [41,42]. If Mus81 was involved in HBV entry but not cccDNA formation, it would also hinder HDV infection. However, HDV-infected Mus81 K.O. cells exhibited similar levels of HDδAg immunofluorescence signals compared to HDV-infected control K.O. cells (S6 Fig), indicating that Mus81 is not involved in HBV/HDV entry but rather serves as a critical factor in *de novo* HBV cccDNA formation.

### Mus81 specifically cleaves 3’ flap on HBV rcDNA substrate *in vitro*

**Fig 7:**
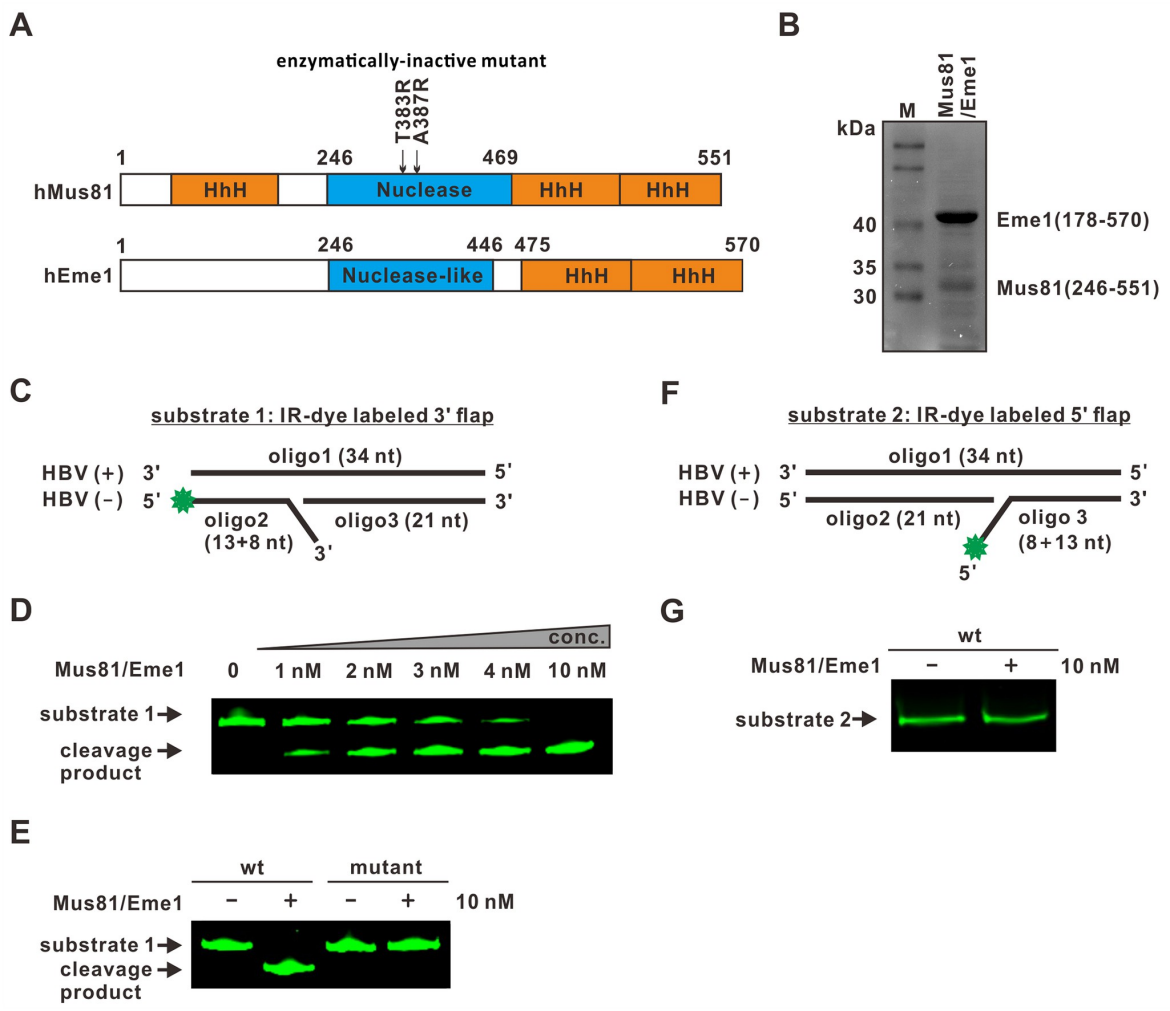
Mus81 specifically cleaves TR in a 3’ flap configuration *in vitro*. (A) Schematic illustration of the structural and catalytic domains of human Mus81 and Eme1. Two Mus81 enzymatically inactive mutations (T383R and A387R) are indicated. HhH: helix-hairpin-helix domain. (B) The Mus81 (aa 246–551)/Eme1 (aa 178–570) enzymatic domain complex was expressed in *E*. *coli*., purified, and verified by SDS-PAGE. (C) Schematic presentation of the fluorescence-labeled synthetic HBV 3’ TR-flap DNA substrate (referred as substrate 1). The green star indicates the fluorescent label. (D) DNA substrate 1 was left untreated or treated with varying concentrations of Mus81-Eme1, followed by native acrylamide gel separation and fluorescence image scanning. (E) DNA substrate 1 was left untreated or treated with wt or mutant Mus81 (T383R+A387R)-Eme1 and subjected to in-gel fluorescence analysis. (F) Schematic presentation of the fluorescence-labeled synthetic HBV 5’ TR-flap DNA substrate (referred as substrate 2). (G) DNA substrate 2 was left untreated or treated with wt Mus81-Eme1, followed by in-gel fluorescence analysis.

To further assess whether Mus81 could remove the 3’ TR of HBV rcDNA in a 3’ flap configuration, we conducted an *in vitro* flap endonuclease cleavage assay. The enzymatically active human Mus81-Eme1 heterodimeric complex was expressed and purified from bacteria, with the enzymatically inactive Mus81 mutant (T383R/A387R) serving as a negative control (Fig 7A and 7B). Then the synthetic short HBV DNA oligos were assembled into dsDNA substrate with either a 3’ flap or 5’flap to mimic the sequence and potential topology of rcDNA at the (-) strand nick region (Fig 7C and 7F). Upon treatment of the HBV DNA substrate with a 3’ flap using the wt Mus81-Eme1 enzyme complex, the fluorescence-labeled substrate exhibited enzyme dose-dependent cleavage, as evidenced by electrophoretic separation of the substrate and cleavage product (Fig 7D). While the 3’ flap substrate was completely cleaved by 10 nM of wt Mus81-Eme1, it was entirely resistant to the enzymatically inactive mutant Mus81-Eme1 at the same concentration (Fig 7E). Furthermore, the wt Mu81-Eme1 failed to digest the DNA substrate with a 5’ flap structure (Fig 7F and 7G). These results demonstrated the specificity of Mus81-mediated cleavage of rcDNA with 3’ TR being positioned as a flap structure.

### Assessing the role of 5’ flap endonucleases in cccDNA formation

**Fig 8:**
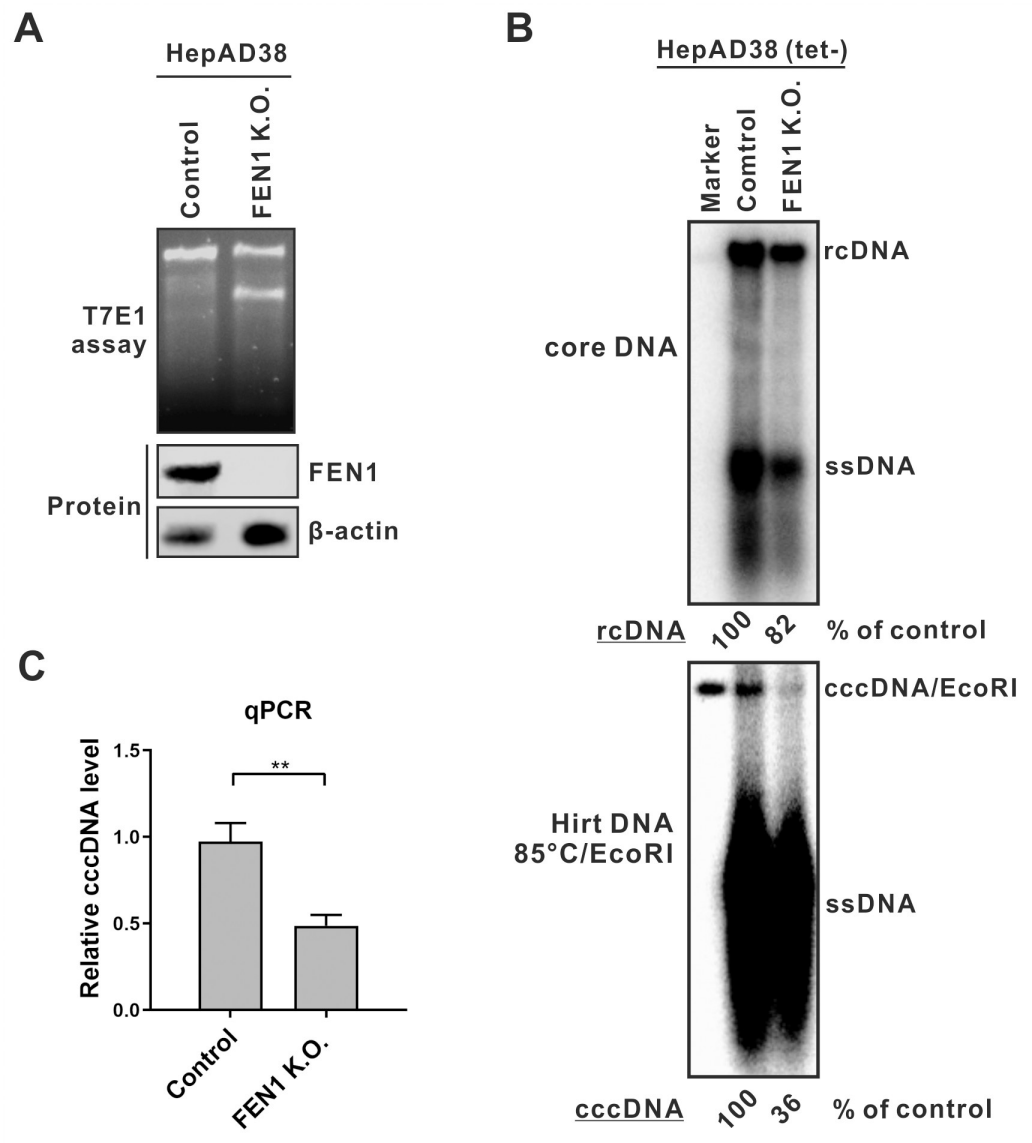
Knockout of FEN1 reduces HBV cccDNA production. (A) Verification of FEN1 knockout. FEN1 gene editing and protein expression in HepAD38 control and FEN1 K.O. cells were analyzed by T7E1 assay and Western blot, respectively. (B-C) HepAD38 control and FEN1 K.O. cells were induced in tet-free medium for 14 days, followed by (B) HBV core DNA and cccDNA Southern blot analyses and (C) cccDNA qPCR assay (mean ± SD, n = 3; **p<0.01).

Previously, the 5’ flap endonuclease FEN1 has been implicated in HBV cccDNA formation [34,35]. However, our mutagenesis results suggest the presence of a 3’ flap-like TR region on rcDNA and subsequent removal of 3’ TR during cccDNA formation, at least for a vast majority of viral DNA populations (Figs 2 and 3). Nonetheless, the incomplete inhibition of cccDNA formation upon Mus81 knockout indicates that other mechanisms may operate in parallel or alternatively. Thus, we revisited the role of FEN1 in cccDNA *via* knocking out FEN1 in HepAD38 cells. The successful knockout of FEN1 was confirmed by T7E1 assay and Western blot (Fig 8A), and by indel sequencing at the genomic DNA level (S7 Fig). We then assessed HBV replication and cccDNA formation levels by Southern blot analysis. Compared to control cells, FEN1 K.O. cells showed a slight reduction of cytoplasmic rcDNA level, but a marked reduction of cccDNA level; after normalizing to rcDNA, the level of cccDNA in FEN1 K.O. cells was reduced to 44% of the control (Fig 8B), which is consistent with previously published data [34]. In the qPCR assay, the cccDNA levels in FEN1 K.O. cells were reduced to approximately 50% of controls after normalizing to the total HBV Hirt DNA levels (Fig 8C). Collectively, these data confirmed that FEN1 does play a role in HBV rcDNA recycling pathway-mediated cccDNA formation as previously reported [34].

**Fig 9:**
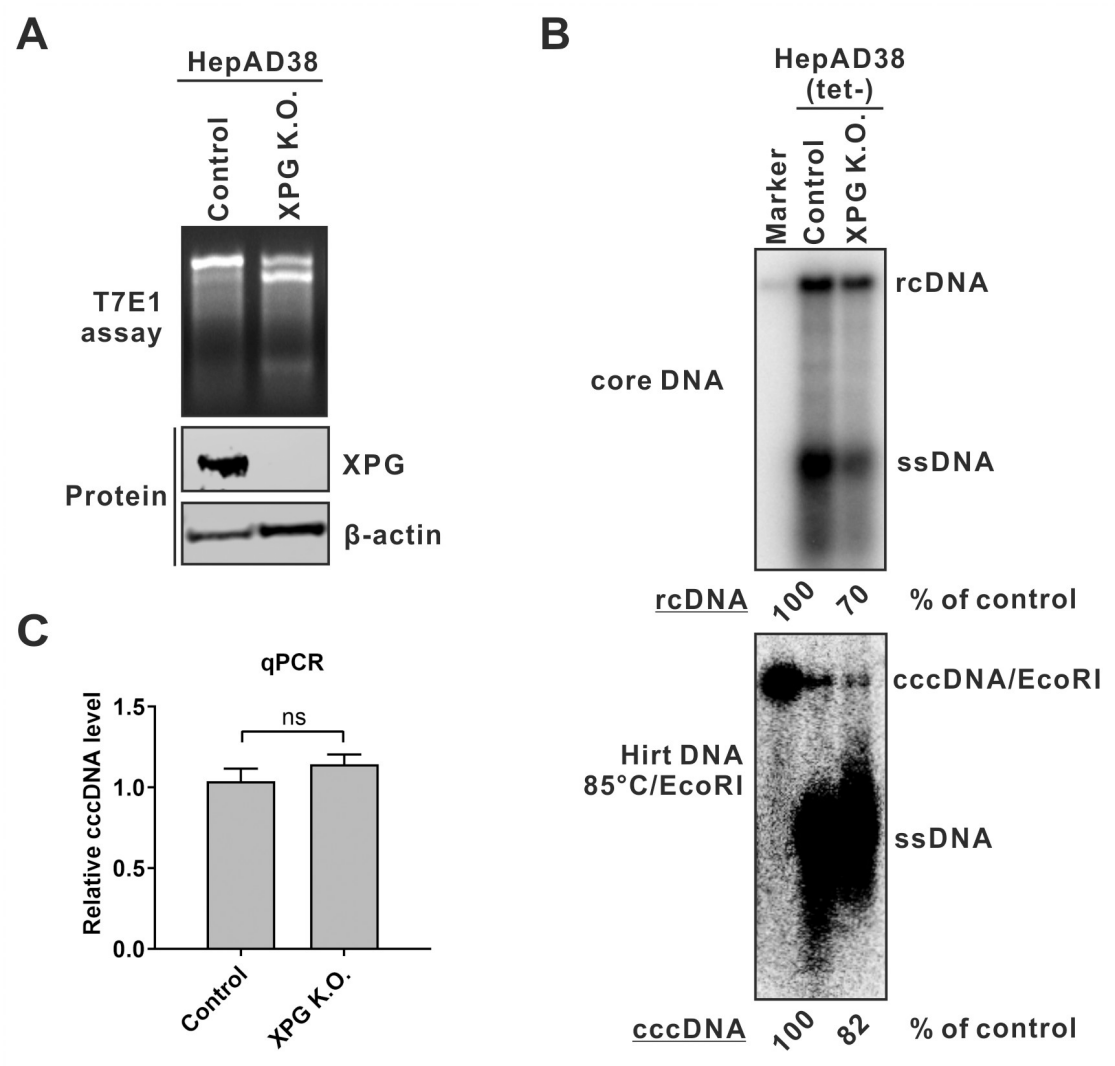
Knockout of XPG has no effect on HBV cccDNA formation. (A) Verification of XPG knockout. XPG gene editing and protein expression in HepAD38 control and XPG K.O. cells were analyzed by T7E1 assay and Western blot, respectively. (B-C) HepAD38 control and XPG K.O. cells were induced in tet-free medium for 14 days, followed by (B) HBV core DNA and cccDNA Southern blot analyses and (C) cccDNA qPCR assay (mean ± SD, n = 3; ns: not significant).

In addition to FEN1, another 5’ flap endonuclease, the human xeroderma pigmentosum group G-complementing protein (XPG), is known to participate in DNA replication damage repair within host cells [43]. Both FEN1 and XPG belong to the FEN1 family of structure-specific nucleases and share a conserved active site. While FEN1 plays a central role in eukaryotic genome DNA lagging-strand replication, XPG is involved in nucleotide excision repair (NER) [43,44]. To explore the potential involvement of XPG in HBV cccDNA formation, we employed CRISPR to knockout XPG in HepAD38 cells (Figs 9A and S8). Southern blot analysis revealed a slight reduction in HBV rcDNA levels in XPG K.O. cells compared to control cells. However, the cccDNA level was proportionally reduced in XPG knockout cells (Fig 9B), and the normalized cccDNA level relative to rcDNA aligns with the cccDNA qPCR result (Fig 9C). These findings ruled out a notable role of XPG in HBV cccDNA formation.

### Mus81 and FEN1 are involved in the formation of CM-rcDNA

**Fig 10:**
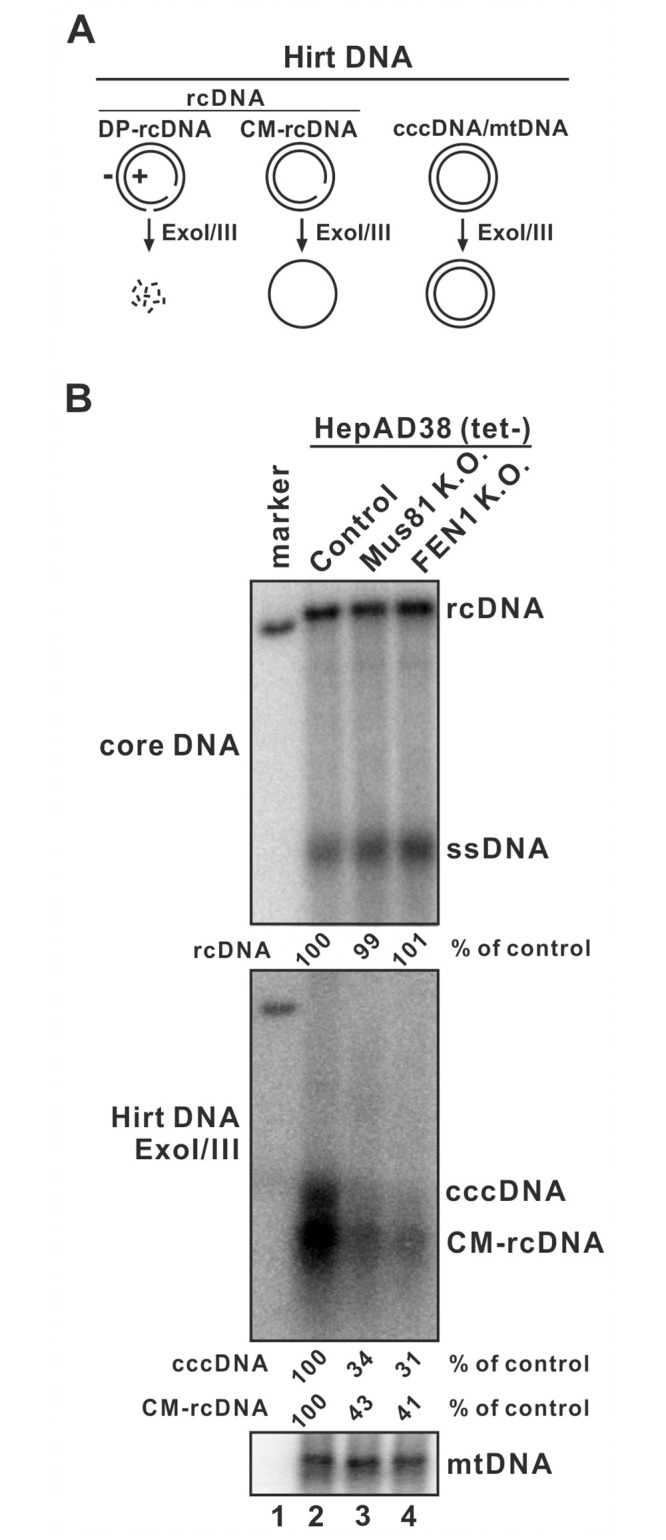
Knockout of Mus81 or FEN1 reduced CM-rcDNA production. (A) Diagram of HBV DNA species and host mitochondrial DNA (mtDNA) in Hirt DNA samples and their remaining products after ExoI/III digestion. DP-rcDNA can be completely removed by ExoI/IIII. CM-rcDNA will become a single-stranded circle (-) strand DNA after ExoI/III treatment. HBV cccDNA and host mtDNA will survive ExoI/III digestion. (B) HepAD38 control, Mus81, and FEN1 K.O. cells were induced in tet-free medium for 10 days. The harvested cells were subjected to HBV cytoplasmic core DNA and total cellular Hirt DNA extraction, and Hirt DNA samples were further treated with ExoI/III, followed by Southern blot analyses.

The CM-rcDNA is a putative intermediate during the conversion of rcDNA into cccDNA [32,33,45,46]. It is anticipated that the removal of one copy of TR from rcDNA is required for synthesizing CM-rcDNA. We then assessed the effect of Mus81 or FEN1 knockout on CM-rcDNA production. Because the CM-rcDNA migrates together with the canonical DP-rcDNA in electrophoresis of Hirt DNA, to visualize CM-rcDNA, 3’ exonucleases ExoI/III are employed to remove DP-rcDNA and the open-ended (+) strand DNA of CM-rcDNA, leaving cccDNA and the circular (-) strand DNA for Southern blot detection according to previous studies [32,45,46] (Fig 10A). As shown in Fig 10B, both the CM-rcDNA and cccDNA were significantly reduced in HepAD38 Mus81 K.O. and FEN1 K.O. cells compared to the control cells, suggesting that Mus81 and FEN1 repair the termini of (-) strand DNA prior to the formation of CM-rcDNA and subsequent cccDNA.

### Mus81 and FEN1 are involved in HBV cccDNA formation in primary human hepatocytes (PHHs)

**Fig 11:**
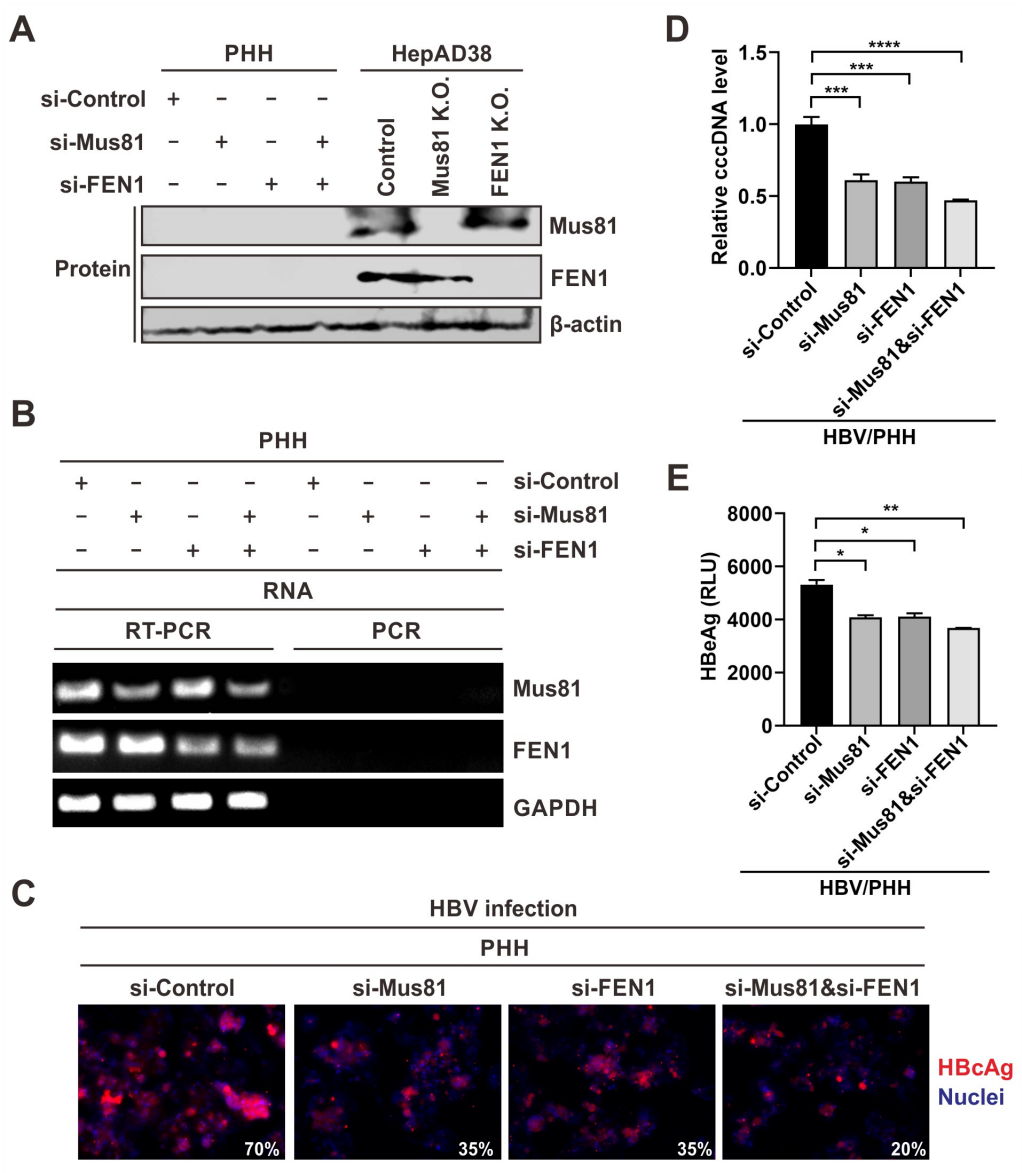
Mus81 and FEN1 are involved in HBV infection in PHH cells. PHH cells seeded in 6-well plate were transfected with 100 pmol of si-control, si-Mus81, si-FEN1, or si-Mus81&si-FEN1 (100 pmol each) for 2 days, followed by HBV infection (500 MOI) for additional 3 days. The harvested PHH cells were subjected to analyses as follows. (A) Western blot of Mus81, FEN1 and loading control β-actin. HepAD38 control, Mus81, and FEN1 KO cell samples served as controls. (B) RT-PCR detection of Mus81, FEN1, and GAPDH mRNA. PCR without the prior RT step served as control to rule out a possibility of genomic DNA contamination. (C) Immunofluorescence of intracellular HBcAg. Cell nuclei were counter staining by DAPI. The percentage of HBcAg-positive cells is indicated. (D) qPCR of intracellular cccDNA. The relative levels of cccDNA were plotted (mean±SD, n = 3; ***p<0.001, ****p<0.0001). (E) Supernatant HBeAg CLIA (mean±SD, n = 2; *p<0.05, **p<0.01).

All the above in-cell studies were conducted in human hepatoma cell line HepG2-derived HBV replication or infection systems, which might possess an aberrant DNA repair machinery due to their cancer cell background. In this regard, we set out to assess the role of Mus81 and FEN1 in cccDNA formation in the physiologically relevant PHH cells. First, we compared the Mus81 and FEN1 expression levels between PHHs and HepAD38 cells (control K.O., Mus81 K.O., and FEN1 K.O.). Interestingly, both proteins were undetectable from PHH cells by Western blot (Fig 11A). While the batch-to-batch variation of PHH and/or antibody sensitivity might affect the Western blot detection, the results indicate that Mus81 and FEN1 may be upregulated in hepatoma cells due to the high rate of cancer cell proliferation and DNA damage compared to the nondividing PHHs [47–51], although down-regulation of Mus81 in hepatoma has also been reported [52]. Nonetheless, the expression of Mus81 and FEN1 in PHHs could still be detected by RT-PCR at the mRNA level (Fig 11B). To assess the function of Mus81 and FEN1 in supporting HBV cccDNA formation in PHHs, we conducted siRNA knockdown of each protein or both, followed by HBV infection. As shown in Fig 11B–11E, the successful knockdown of each target protein in PHHs markedly inhibited HBV infection as measured by intracellular cccDNA qPCR, HBcAg immunofluorescence, and supernatant HBeAg ELISA; furthermore, the double knockdown of both Mus81 and FEN1 exhibited further inhibition of HBV infection. These results suggest that Mus81 and FEN1 play an efficient cooperative role in cccDNA formation in PHH cells.

### Mapping the FEN1 and Mus81 cleavage sites in TR by RACE-NGS

We have previously reported that the cytoplasmic HBV rcDNA and DP-rcDNA possess both copies of full-length TR sequence on the (-) strand DNA [31], indicating that further processing of TR occurs after nuclear import of rcDNA. To reveal the landscape of TR processing in cell nucleus, the nuclear Hirt DNA (predominantly DP-rcDNA) were isolated from induced HepAD38 control, FEN1 K.O., and Mus81 K.O. cells, and subjected to 5’ and 3’ RACE reactions of the (-) strand DNA, followed by NGS and bioinformatic analyses.

**Fig 12:**
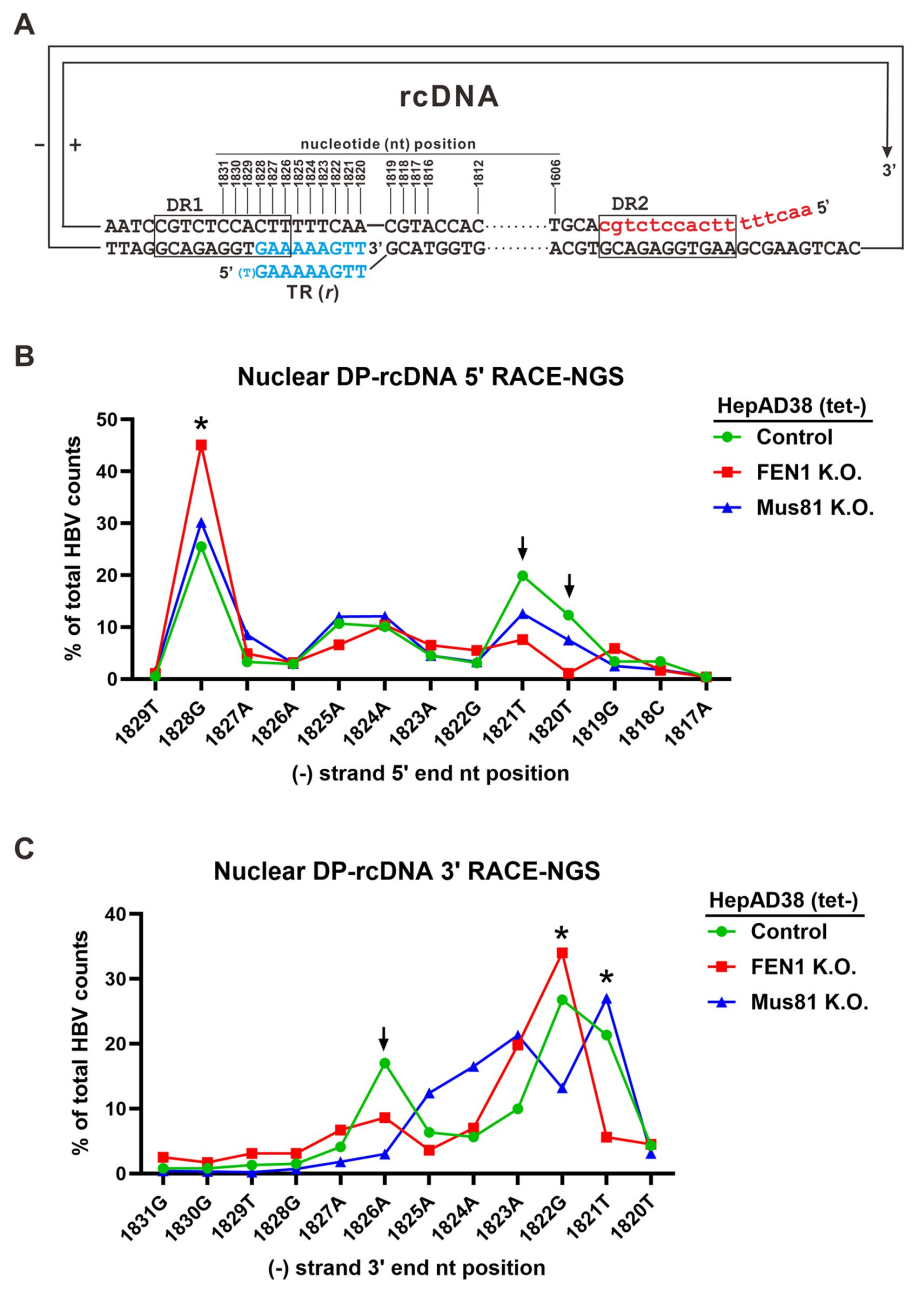
Identification of potential FEN1 and Mus81 cleavage sites in TR by RACE-NGS. (A) Schematic illustration of the cohesive region of HBV rcDNA. The (+) and (-) strands of rcDNA and their 5’ and 3’ ends are denoted. The TR (*r*) nucleotide sequences of (-) strand DNA are shown in blue. For convenience, the 3’ TR is shown as complementary with (+) strand DNA. The bracketed small T indicates the noncanonical first primed nucleotide 1829T at the 5’ end of (-) strand DNA. The nucleotide (nt) positions of genotype D HBV DNA are indicated according to the Galibert nomenclature [78]. DR1 and DR2 regions are boxed. The parallel dotted lines represent the omitted internal DNA sequences between TR and DR2. The 5’ RNA primer sequences are shown in red lowercase letters, the tilted sequences indicate the 5’ uncomplementary portion. (B-C) The nuclear DP-rcDNA was extracted from induced HepAD38 control, FEN1, and Mus81 K.O. cells and subjected to (-) strand DNA 5’ and 3’ RACE-NGS assay, followed by bioinformatic analyses to identify the terminal nucleotide within the TR sequence. The percentage of the number of each full-length and truncated 5’ and 3’ end, relative to the total 5’ and 3’ TR RACE-NGS counts for each sample, was plotted, respectively. The Y-axis represents the terminal nucleotide of identified TR with varying lengths. The terminal nucleotide of full-length 5’ and 3’ TR is marked with an asterisk. Arrows indicate the cleavage sites for FEN1 (B) and Mus81 (C).

As shown in Fig 12A and 12B and S1 Table, the 5’ termini of nuclear DP-rcDNA (-) strand from the control cells were predominantly mapped at 1828G (indicated by an asterisk), which corresponds to the major priming site of (-) strand DNA synthesis; two major putative 5’ TR cleavage sites were mapped at 1821T and 1820T (marked by down arrows), indicating the formation of 5’ flap by the full-length 5’ TR and cleavage by FEN1. In line with this, the frequencies of nuclear DP-rcDNA with terminal 1821/1820T was markedly reduced in FEN1 K.O. cells, together with an increase of terminal 1828G frequency. Knockout of Mus81 resulted in a modest reduction of terminal 1821/1820T frequencies on DP-rcDNA, inferring a potential effect of Mus81 knockout on FEN1-mediated TR cleavage (see Discussion).

Regarding the 3’ end of nuclear DP-rcDNA (-) strand, the longer 3’ termini in induced HepAD38 control cells were predominantly mapped at 1821T and 1822G (Fig 12A and 12C and S1 Table) (indicated by asterisks), while the former position is consistent with the 3’ RACE result of rcDNA (-) strand from HepDES19 cells (Fig 2) [31], representing the unprocessed 3’ end; the latter might be converted from pgRNA initiated at 1822C and selectively detected by RACE-NGS or resulted from one nucleotide (1821T) excision from the 3’ end of DP-rcDNA (-) strand in nucleus. The underrepresented canonical 3’ terminal 1820T of rcDNA (-) strand in HepAD38 and HepDES19 cells is due to the tet-CMV promoter-driven pgRNA transcription from the engineered HBV transgene in those inducible HBV stable cell lines [26,31,53]. The majority of recessed 3’ ends was mapped at 1826A in control cells (marked by a down arrow), which was significantly diminished upon knockout of Mus81, indicating that Mus81 may predominantly cleave the 3’ TR between 1826A and 1825A. Additionally, there was a reduction of terminal 1822G in Mus81 K.O. cells compared to control cells, but this is unlikely a Mus81 cleavage site due to its adjacency to the 3’ end of full-length TR. In FEN1 K.O. cells, there was a moderate reduction of 1826A at 3’ end of the (-) strand of nuclear DP-rcDNA, but more strikingly, a significant loss of 1821T and an increase of 1822G at the 3’ end. Again, the reason for the observed 3’ 1-nt recession of nuclear DP-rcDNA (-) strand in HepAD38 control and FEN1 K.O. cells is unknown, but it may be catalyzed by a cellular 3’ exonuclease under such circumstances.

It is worth noting that, although the putative TR cleavage sites by FEN1 and Mus81 were identified, both 5’ and 3’ TR exhibited heterogeneous lengths (Fig 12B and 12C and S1 Table), indicating that the TR sequences of nuclear DP-rcDNA undergo various trimming processes and generate heterogeneous intermediates and even dead-end products, which may explain the observed accumulation of excess DP-rcDNA in HBV stable cell lines in this and other studies [26,27,54].

## Discussion

The establishment and persistence of HBV infection rely on viral cccDNA, which is a non-replicating episomal genome formed in the nucleus of infected hepatocyte through conformational conversion of the viral rcDNA [1]. To compensate for its limited gene-coding capacity, HBV hijacks host functions to complete its life cycle [14]. Cellular DNA repair is a well-conserved system that monitors and repairs damage in chromosomal DNA, maintaining the stability and integrity of the host genome for replication and transcription [55,56]. It is now well acknowledged that HBV exploits the cellular DNA repair machinery for cccDNA formation by mimicking the rcDNA as a "damaged" DNA. The gap and nick present on rcDNA may be identified as DNA damage by the host DNA repair system, and the rcDNA termini and associated modifications are likely to undergo a series of substrate-specific processing, including removal of Pol and RNA primer, DNA trimming, elongation, and ligation during cccDNA formation. Among these processes, one copy of the TR on rcDNA (-) strand needs to be removed to maintain the unit length of HBV genome sequence on cccDNA (Fig 1A). Based on the rcDNA genomic organization, it is conceivable that the TR region may display a flap structure, however, it remains unclear whether a 5’ or 3’ flap is formed, which could be originally determined by which copy of TR is used as the template for (+) strand DNA synthesis during viral DNA biosynthesis (Fig 1B).

Previous studies have attempted to address these questions using the DHBV model [24,57,58]. While the identical sequence of DHBV TR is not necessary for template switch during (+) strand DNA elongation and either copy of TR can be copied into (+) strand sequence, it does prefer AT-rich TR sequence for efficient rcDNA circularization [57,58]. Consistent with this, HBV TR is also AT-rich (Fig 1B). A later study introduced mutation into the 5’ TR of DHBV rcDNA and demonstrated that the mutated 5’ TR was preferentially used as template for (+) strand synthesis (~85% frequency) but the mutation was significantly replaced by wt sequence on cccDNA (36–78% frequency), indicating a preferential removal of mutant 5’ TR during DHBV cccDNA formation in chicken hepatoma LMH cells [24]. In line with this, a 5’ flap endonuclease FEN1 has been shown to cleave the 5’ flap on synthetic HBV rcDNA substrates, and knockdown of FEN1 partially reduced HBV cccDNA formation in HepAD38 cells [34,35]. The observed heterogeneous DHBV cccDNA sequences in the DHBV TR mutagenesis study and the incomplete inhibition of HBV cccDNA production upon FEN1 inhibition indicate that alternative cellular nuclease(s) and/or viral rcDNA TR topologies exist in cccDNA formation.

Considering the potential viral and host discrepancies between DHBV and HBV cccDNA formation, we specifically investigated the selection of TR during the synthesis of HBV (+) strand DNA and cccDNA in the present study. To do that, we took a different approach from the previous DHBV mutagenesis study by introducing a point mutation (G1822C) into the 3’ TR instead of 5’ TR to avoid aberrant priming of (-) strand DNA (Fig 2A and 2D) [24,59]. The mutant viral genome replicated rcDNA and cccDNA as efficiently as wt in stably transfected HepDES-C1822G cells (Fig 2B and 2C). In addition, we utilized NGS approach to significantly increase the coverage of TR sequence populations compared to the conventional clone sequencing. Interestingly, the PCR-NGS results demonstrated that both viral (+) strand DNA and cccDNA in HepDES-C1822G cells predominantly harbor the wt TR sequence (Figs 3 and S1), suggesting that the 5’ TR serves as the template for (+) strand elongation and the 3’ TR is removed during the process of cccDNA formation. While these results are partly consistent with the DHBV study, especially the 5’ TR selection during rcDNA formation regardless of whether it is wt or mutant; the almost complete absence of mutated 3’ TR sequence on cccDNA indicates certain virus- or host-specific mechanism(s) governing HBV and DHBV cccDNA formation. However, it is also possible that hepadnaviruses could selectively get rid of the mutated TR moiety as a quality control mechanism. Nonetheless, these results indicated DNA flap structure might be formed at TR region of HBV rcDNA and inspired us to further explore the role of cellular 5’ and 3’ flap endonucleases in cccDNA formation.

Structure-specific endonucleases (SSEs) process DNA secondary structures that arise during DNA replication, DNA repair, DNA recombination and transcription [43]. Mammalian DNase IV enzymes, including FEN1 and XPG (also known as ERCC5), have been demonstrated to exhibit similar specificity for DNA structures rather than sequences; both act with defined polarity by specifically cutting the strand that forms a 5’ flap at double-stranded/single-stranded (ds-ss) junctions [60,61]. The heterodimeric XPF-ERCC1 complex functions as 3’ flap endonucleases, acting with polarity opposite to that of FEN1 and XPG. [62,63]. Additionally, SSEs that are capable of processing Holliday junctions have been identified and named Holliday junction resolvases. Holliday junction resolvases were unexpectedly all found to be flap endonucleases. The first of these to be discovered were the fission yeast and human Mus81-Eme1 endonucleases, which are closely related to the XPF-ERCC1 endonuclease but possess the additional ability to process more complex branched DNA structures [64,65]. In general, XPF and Mus81 function as 3’ flap endonucleases, while FEN1 and XPG act as 5’ flap endonucleases.

Based on the TR mutagenesis and NGS results showing an absence of mutated 3’ TR on cccDNA (Fig 3), we first assessed the potential role of 3’ flap endonucleases in cccDNA formation. Knockout of Mus81 but not XPF in induced HepAD38 cells significantly reduced HBV cccDNA production (~70% reduction) without affecting cytoplasmic core DNA replication, and the results were consistent among multiple knockout cell clones (Figs 4, 5 and S2–S4), suggesting that Mus81 is involved in rcDNA recycling-mediated cccDNA formation. Furthermore, Mus81 appears to also participate in the *de novo* cccDNA formation from infecting virus, as evidenced by that knockout of Mus81 in HepG2-NTCP cells markedly reduced the levels of cccDNA and viral antigen expression upon HBV infection without affecting the viral entry, and that ectopic expression of Mus81 rescued cccDNA and antigen production in HepG2-NTCP Mus81 K.O. cells (Figs 6, S5 and S6). These results indicate that Mus81 plays a critical role in HBV cccDNA presumably *via* removing the 3’ TR of rcDNA.

Mus81 is known to partner with Eme1 and process the stalled replication forks before they regress to form a Holliday junction. This Mus81-Eme1 complex prefers cleaving 3’ flap DNA substrates with 5’ nicked ends [40], which is reminiscent of one potential rcDNA topology at the TR region (Fig 1B). In an *in vitro* biochemical reaction, Mus81-Eme1 duet was able to specifically resolve the flap structure formed by 3’ TR but not 5’ TR on a synthetic rcDNA fragment in an endonuclease activity-dependent manner (Fig 7), further confirming the ability of Mus81 to remove 3’ TR if it forms a 3’ flap structure on rcDNA.

The incomplete inhibition of cccDNA formation in the absence of Mus81 suggests that other cellular DNA nuclease(s) and/or HBV TR structure(s) may exist in cccDNA formation (Figs 5 and 6). Thus, we further assessed the two known cellular 5’ flap endonucleases, specifically FEN1 and XPG, for their potential roles in cccDNA formation. While knockout of XPG showed no obvious reduction of cccDNA formation in HepAD38 cells (Figs 9 and S8), the loss of FEN1 resulted in a marked inhibition of cccDNA production (Figs 8 and S7), indicating an involvement of FEN1 in cccDNA formation, which is consistent with a previous study [34]. Furthermore, knockout of either Mus81 or FEN1 in HepAD38 cells markedly reduced the level of CM-rcDNA (Fig 10), a putative intermediate during rcDNA to cccDNA conversion that also requires the removal of one copy of TR from rcDNA before close up of the (-) strand DNA [32].

We attempted to knock out both Mus81 and FEN1 by CRISPR but was unsuccessful, likely due to the requirement for at least one of these two flap endonucleases being present to maintain cell viability, and hence, although we have assessed all the four known flap endonucleases for their involvement in cccDNA formation, it remains possible that there is an unidentified flap endonuclease(s) plays redundant role in HBV rcDNA TR removal. On the other hand, we demonstrated that siRNA-mediated knockdown of either Mus81 or FEN1 in PHH cells inhibited *de novo* HBV infection, including cccDNA formation; this inhibition was further enhanced by the double knockdown, confirming their cooperative role in cccDNA formation in the physiologically-relevant natural host cells for HBV infection (Fig 11). Interestingly, both enzymes were expressed at much lower levels in PHHs compared to HepG2 hepatoma cell line (Fig 11A). Given the potential differences of DNA repair machinery between normal and cancerous hepatocytes, it is crucial to validate the cccDNA-related cellular DNA repair factors initially identified in hepatocyte-derived cancer cell line systems using PHH cells, ideally from multiple batches and different donors, in this type of studies.

The results presented in this study clearly demonstrate that Mus81 and FEN1 exert an overlapping activity in cccDNA formation, which also indicates that both the 3’ and 5’ TR-derived flap structures are existing on rcDNA to serve as substrates for Mus81 and FEN1, respectively, during cccDNA formation. While the (+) strand DNA sequencing results suggest that a DNA duplex forms between the 5’ TR and the (+) strand during rcDNA formation, leaving the 3’ TR as a flap (Figs 1B and 3A), it is also plausible that a dynamic flap structure forms at both ends of the (-) strand after template switching during (+) strand elongation or after the deproteination of polymerase from rcDNA, or following the nuclear import of DP-rcDNA [25–27,31]. This possibility is particularly relevant given that the AT-rich TR (8–9 nt) is short enough to allow interchangeable annealing between the 5’ and 3’ TR with complementary (+) strand DNA due to its low melting temperature (Tm), or cellular DNA helicases are involved in regulating the TR-flap dynamics. However, it remains unknown whether FEN1 and Mus81 cleave a single rcDNA molecule simultaneously or sequentially if the dynamic flap structure can form on both ends of (-) strand DNA.

Our previous analyses of the termini of cytoplasmic rcDNA and DP-rcDNA revealed that both the 5’ and 3’ TR maintain integrity, indicating that the trimming of TR occurs in the nucleus after the nuclear import of rcDNA [31]. In this study, we further sequenced the termini of nuclear DP-rcDNA (-) strand by RACE-NGS. The sequence analyses revealed two potential FEN1 cleavage sites (1821T and 1820T) within the 5’ TR and one potential Mus81 cutting site (1826A) within the 3’ TR (Fig 12 and S1 Table), further suggesting that both 5’ and 3’ TR-flap structures exist on nuclear rcDNA for FEN1 and Mus81 to target, respectively. While the mapped FEN1 cleavage site indicates a complete removal of 5’ TR; Mus81 reaction still leaves 2 nt of 3’ TR being uncleaved, indicating that further processing of 3’ and/or 5’ TR is required to generate unit-length (-) strand DNA for CM-rcDNA and cccDNA formation. In line with this, many minor nuclear DP-rcDNA species with heterogeneous lengths of 5’ and 3 TR have been identified by the RACE-NGS assay (Fig 12 and S1 Table), which may be the further intermediates or dead-end products during cccDNA formation. Additionally, although nuclear DP-rcDNA was obtained by gel extraction and purification for RACE-NGS, we cannot completely rule out a possible minor contamination by the DP-dslDNA species co-existing in the nucleus of HBV replicating cells. In contrast with rcDNA, the (-) strand DNA TR sequences of dslDNA are annealed with both ends of (+) strand DNA and undergo trimming by the error-prone non-homologous end joining (NHEJ) repair machinery for intramolecular circularization (cccDNA with indels) or integration into host chromosomal DNA [19,66].

**Fig 13:**
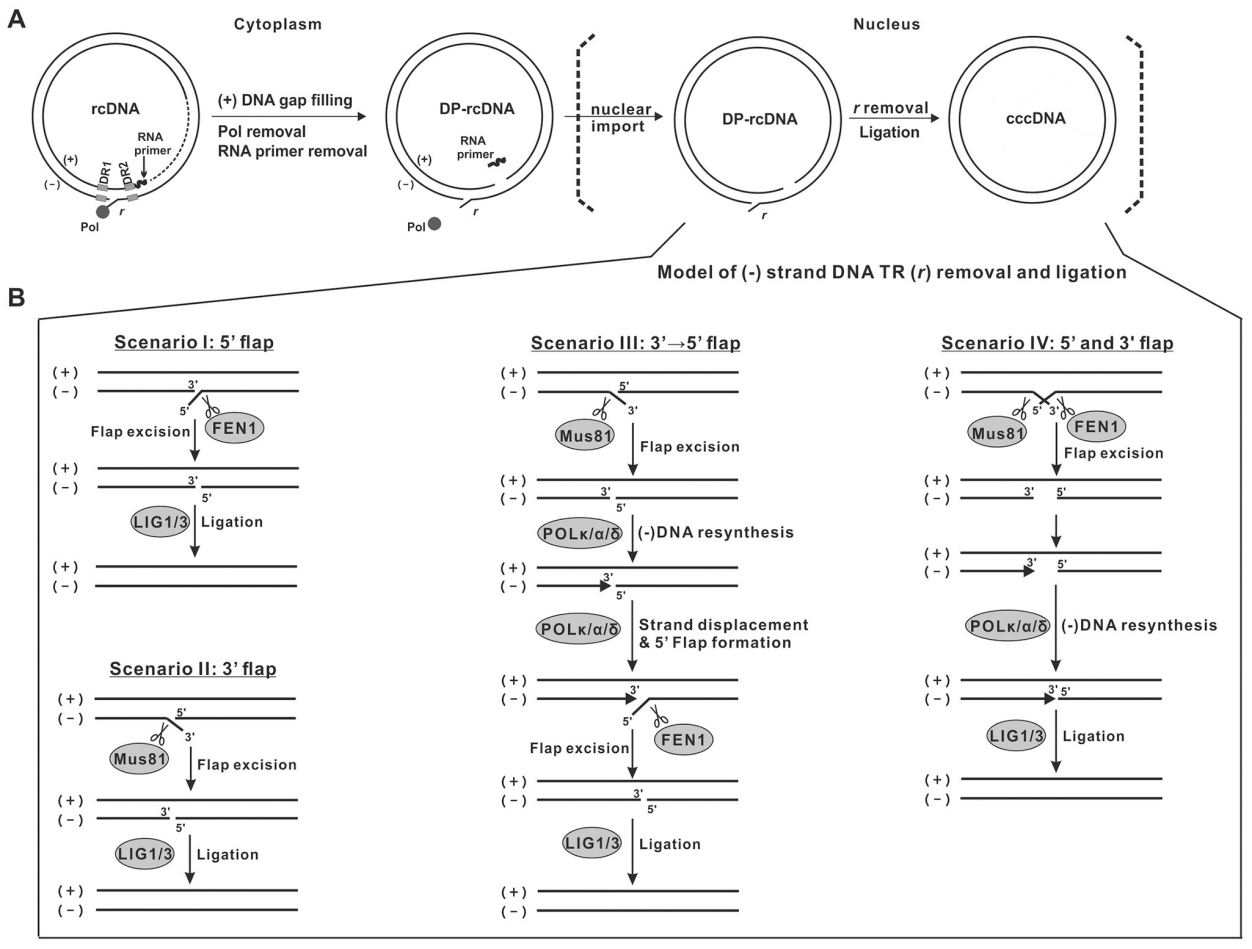
Model of HBV rcDNA TR processing during cccDNA formation. (A) Diagram of a proposed molecular pathway of cccDNA formation with DP-rcDNA as an intermediate. For convenience, the 5’ TR (*r*) on the (-) strand of rcDNA/DP-rcDNA is depicted as a flap structure. In the cytoplasm, the gap filling of (+) strand DNA of rcDNA triggers the removal of viral RNA primer from 5’ end of (+) strand and Pol from 5’ end of (-) strand DNA, giving rise to the cytoplasmic DP-rcDNA, followed by import of DP-rcDNA into nucleus, where the cellular DNA repair machinery removes one copy of the TR on (-) strand DNA of DP-rcDNA and prepares the termini of both DNA strands for ligation into cccDNA [19,25–27,31]. (B) The proposed model of TR processing on the (-) strand DNA of nuclear DP-rcDNA presents four major possible scenarios, depending on the different polarities of the flap structure formed by TR and the timing of FEN1 and Mus81 involvement. Refer to the text for a detailed description.

Taken together, we propose herein a model for HBV rcDNA TR processing during cccDNA formation. Upon the entry of DP-rcDNA into the nucleus, the host DNA repair and replication factors are utilized to remove one copy of the TR (Fig 13A). The putative 5’ and 3’ TR-flaps are dynamically interchangeable, and FEN1 and Mus81 act as 5’ and 3’ TR-flap endonucleases, respectively. There are four possible scenarios (Fig 13B): **I)** the full-length 5’ TR (nt 1828–1821) exhibits as a flap, FEN1 directly removes the entire 5’ flap structure, and subsequently, the 3’ TR annealed with (+) strand DNA is ligated with the processed 5’ end to close up the (-) strand DNA by ligase 1 and/or 3 (LIG1/3) [35,36]. **II)** rcDNA processes a 3’ TR-flap, Mus81 removes a large portion of the 3’ flap (nt 1826–1821) first, followed by further 2-nt trimming of the 3’ TR by undefined nuclease(s) to completely remove the 3’ TR for (-) strand DNA ligation. In this scenario, the 2-nt trimming must be rapidly coupled with ligation, as no major 3’ end at nt 1828 was identified by RACE-NGS (Fig 12C). **III)** after 3’ TR removal as described in scenario II, host DNA polymerases (POLκ, α, δ) [35,45,67] are recruited to the gap and resynthesize the 3’ TR using (+) strand DNA as template, then the continued DNA elongation displaces the 5’ TR, forming a 5’ flap for FEN1 to cut as shown in scenario I. **IV)** both 5’ and 3’ flap structures are formed on the same nuclear DP-rcDNA molecule and simultaneously removed by FEN1 and Mus81, respectively, followed by 3’ TR resynthesis and ligation. In this scenario, further removal of nt 1828–1827 dinucleotide is not needed after Mus81 cleavage. While all the above four possible scenarios apply to wt HBV, the 3’ TR cutting-ligation and cutting-resynthesis-ligation models (scenarios II-IV) can also explain why the mutated 3’ TR is excluded from the final cccDNA product (Fig 3).

Our study and the proposed model of TR processing by Mus81 and FEN1 during rcDNA to cccDNA conversion further highlight the complex nature of HBV DNA replication and the intricate interplay between host DNA repair machinery and the virus. Growing evidence indicates that HBV cccDNA formation exploits multiple alternative and redundant activities of cellular DNA repair factors and pathways, which is a key feature of cccDNA biosynthesis, contributing to its productivity and persistence during viral infection [19]. This concept should be taken into account when investigating the mechanisms of cccDNA formation and developing host-targeting strategies to inhibit cccDNA biosynthesis.

## Materials and methods

### Cell cultures

HepG2 and 293T cells were cultured in DMEM/F12 medium supplemented with 10% fetal bovine serum, 100 U/ml penicillin and 100 μg/ml streptomycin. The tetracycline (tet)-inducible HBV (genotype D, subtype *ayw*) stable cell lines HepDES19 and HepAD38 were described previously [26,53], and were maintained in the same culture condition for HepG2 cells with an additional 1 μg/ml of tet and 400 μg/ml of G418. When required, the culture medium was switched to tet-free to induce HBV replication in HepAD38 or HepDES19 cells. HepG2-NTCP cells were described previously and cultured in the same medium for HepG2 cells with an additional 8 μg/ml of blasticidin. Freshly isolated PHH cells were obtained through the Human Liver Tissue and Hepatocyte Research Resource (HLTHRR, funded by NIDDK project# R24DK139775) at The Pittsburgh Liver Research Center (PLRC, funded by NIDDK grant# P30DK120531), University of Pittsburgh, and cultured as previously described [68,69].

### Plasmids, siRNA and transfection

Plasmid pTREHBVDES, the vector delivering the HBV transgene in HepDES19 cells, has been described previously [26]. Plasmid pcFLAG-Mus81 expressing N-terminally Flag-tagged human Mus81 (GenBank Accession No.: BC009999) was purchased from Sino Biological (HG14854-NF). Prokaryotic expression plasmids pCDF-duet-hMus81 (aa 246–551), pCDF-duet-hMus81 (T383R+A387R), and pET-duet-hEme1 (residues 178–570) were the gifts from Dr. Yunje Cho (Pohang University of Science and Technology, South Korea) [40]. Transfection of plasmids into cell cultures was performed using Liofectamine 3000 (ThermoFisher Scientific, L3000150) according to manufacturer’s directions.

The control, Mus81 and FEN1 siRNAs were purchased from Santa Cruz Biotechnology (sc-37007, sc-40751 and sc-37795, respectively). To transfect siRNA into PHH cells, Lipofectamine RNAiMAX Transfection Reagent (ThermoFisher Scientific, 13778100) was used, and the transfection solution was made according to the manufacturer’s manual. After adding the siRNA transfection complex solution onto the plated PHH cells, the plate was spun at 1,000 ×g in a micro plate centrifuge for 1 h at room temperature to enhance the transfection efficiency.

### HBV DNA extraction, Southern blot, and qPCR

The cytoplasmic HBV capsid-associated DNA (core DNA) was extracted as previously described [26]. The protein-free HBV DNA (cccDNA, DP-rcDNA, DP-dslDNA, and CM-rcDNA) was extracted by using a modified Hirt extraction procedure as previously described [26,39,70]. Southern blot analyses of HBV core DNA and Hirt DNA were conducted according to previous publications [26,39]. When indicated, HBV Hirt DNA was heat denatured at 85°C for 5 min, followed by EcoRI digestion for a better separation of the linearized cccDNA and denatured DP-rcDNA/dslDNA/CM-rcDNA on Southern blot [26,39]. For detection of CM-rcDNA, the Hirt DNA sample was digested by exonucleases I and III (ExoI/III) (NEB, M0293S/M0206S) and subjected to Southern blot assay as previously described [32,46]. The qPCR quantifications of total HBV Hirt DNA, cccDNA, and cellular mitochondrial DNA (mtDNA) were performed as previously described [68,71]. For HBV stable cell lines, cccDNA qPCR data was normalized by total Hirt DNA qPCR; in terms of HBV-infected HepG2-NTCP or PHH cells, mtDNA qPCR data was used to normalize cccDNA qPCR.

### Establishment of the tet-inducible HepDES-C1822G HBV stable cell line

A point mutation C1822G was introduced into the 5’ end of HBV sequence in plasmid pTREHBVDES by replacing the DNA fragment between the SacI and BspEI restriction sites with a chemically synthesized DNA fragment containing C1822G mutation, the obtained plasmid was named pTREHBVDES-C1822G and confirmed by Sanger sequencing. Next, HepG2 cells were co-transfected with plasmids pTREHBVDES-C1822G and pTet-off (Takara, 631017) in 7:1 molar ratio using Lipofectamine 3000 (Invitrogen, L3000150). The transfected HepG2 cells were selected by 500 μg/ml of G418 in the presence of 1 μg/ml of tet, and the G418-resistant cell colonies were expanded into cell lines, followed by assessment of tet-inducible HBV replication as previously described [72]. One cell line supporting a similar level of tet-inducible (tet-off) HBV DNA replication with HepDES19 cell line was designated HepDES-C1822G.

### HBV rcDNA (-) strand 3’ RACE

Cytoplasmic HBV core DNA was extracted from the induced HepDES19 and HepDES-C1822G cells, and the rcDNA species was purified and subjected to (-) strand DNA 3’ rapid amplification of cDNA ends (RACE) analysis according to our previous publication with minor modifications [31]. Briefly, approximately 20 pg of HBV rcDNA was denatured at 95°C for 10 min and then kept on ice. The denatured DNA was mixed with 5 μl of 2 × T4 RNA ligase reaction buffer containing an additional 50% PEG-8000, 2 mM hexamine cobalt chloride, 0.5 μl of 50 μM RACE anchor oligo (S2 Table), and 1 μl of T4 RNA ligase 1 (NEB, M0204). Nuclease-free water was supplied to bring the reaction volume to 10 μl. The mixture was incubated at room temperature for overnight. 3 μl of DNA ligation product was used as PCR template to amplify the 3’ end of rcDNA (-) strand using HBV- and RACE anchor-specific primers (S2 Table) and the Advantage HF 2 PCR Kit (Takara, 639123) with the previously described PCR conditions [31]. The PCR product was cloned into pGEM-T vector (Promega, A362A), followed by Sanger sequencing.

### PCR amplification of HBV rcDNA (+) strand TR-complementary sequence and NGS

Cytoplasmic HBV core DNA was extracted from the induced HepDES19 and HepDES-C1822G cells. The amplification of HBV rcDNA (+) strand sequence complementary to the TR region of rcDNA (-) strand contains two steps. First, a chimeric oligo (Rrc) containing 5’ non-HBV sequence and sequence complementary to rcDNA (+) strand were used for primer extension (S1A Fig and S3 Table). The reaction mixture was denatured at 95°C for 5 min, and the amplification was set for 10 cycles of 95°C for 20 s, 65°C for 20 s (-1°C each cycle), and 72°C for 30 s, another 15 cycles of 95°C for 20 s, 55°C for 20 s and 72°C for 30 s, with an addition 5 min extension at 72°C after the final cycle. The amplification product was purified by the High Pure PCR Product Purification Kit (Roche, 11732668001). The above primer extension product was used as template for second step of amplification with oligo Ry and Frc1 (for HepDES19 sample) or Frc2 (for HepDES-C1822G sample) as primers (S1A Fig and S3 Table). The reaction mixture was denatured at 95°C for 5 min, and the amplification was set for 35 cycles of 95°C for 20 s, 60°C for 20 s, and 72°C for 40 s, with an additional 5 min extension at 72°C after the final cycle. The final PCR product was purified for next-generation sequencing (NGS). Quality estimation of raw NGS reads was performed with the fastqc tool, reads with an average quality score lower than 20 were discarded. Quality control passed reads were aligned against the reference HBV genotype D sequence (GenBank Accession No. U95551.1) with local BLASTn using the following paraments: ’-evalue 1e-5, gapopen 10, gapextend 4, penalty 3’. The sequences aligned with the reference sequence were subjected to nucleotide constitution frequency matrix analysis.

### PCR amplification of HBV cccDNA TR sequence and NGS

HBV Hirt DNA samples extracted from the induced HepDES19 and HepDES-C1822G cells were heat denatured at 85°C for 5 min, followed by Plasmid-Safe ATP-Dependent DNase (PSAD) (Bioresearch Technologies, E3101K) treatment overnight to remove non-cccDNA species. After inactivation of PSAD at 70°C for 20 min, cccDNA was purified by using DNA Clean & Concentrator-5 Kit (ZYMO, D4004). The purified cccDNA samples were subjected to PCR using the indicated primers (S1B Fig and S4 Table). The PCR products were purified using QIAquick PCR Purification Kit (Qiagen, 28106) and subjected to NGS analysis as above mentioned.

### Establishment of gene knockout cell lines

The XPF, Mus81, FEN1 and XPG knockout cell lines were generated through CRISPR-mediated genome editing of their gene loci. The single guide (sg) RNAs targeting human XPG, XPF, FEN1 and Mus81 genes were designed at http://chopchop.cbu.uib.no/ and shown in S2, S4, S7 and S8 Figs. In addition to the general criteria for sgRNA design, the sgRNAs were designed to target either the 5’ end of the ORF or the functional domain coding region [73]. Furthermore, the designed sgRNA sequences do not possess any possible CRISPR sites in HBV genome. The synthetic sgRNA oligo pairs (S5 Table) were annealed and cloned into BsmBI-digested lentiCRSPRv2 control vector (Addgene #52961, gift from Dr. Feng Zhang). Lentivirus preparations were performed according to the protocols from Dr. Feng Zhang’s Lab (genome-engineering.org). Briefly, each lentivector was co-transfected with packaging plasmids psPAX2 and pMD2.G (Addgene #12260 and #12259, respectively, gift from Dr. Didier Trono) in molar ratio of 4:3:1 into 293T cells by Lipofectamine 3000, and 48 h post-transfection, medium was collected and filtered through a 0.45 μm filter. Virus titers were determined by the Lenti-X qRT-PCR Titration Kit (Takara, 631235). HepAD38 or HepG2-NTCP cells were transduced by collected lentiviral XPF-, Mus81-, FEN1-, and XPG-sgRNAs or lentiviral control scramble sgRNA. The transduced cells were selected with 2 μg/ml puromycin, and the antibiotics-resistant cells were pooled and expanded into cell lines. The knockout of XPF, Mus81, FEN1, and XPG in each corresponding cell line was assessed by T7 endonuclease I (T7E1) assay [74], indel sequencing, and Western blot.

### T7E1 assay and indel sequencing

The PCR-based T7E1 mismatch detection assay was performed by using the Guide-it Mutation Detection Kit (Takara, 631443) according to manufacturer’s manual. The T7E1 PCR primers amplifying the XPF, Mus81, FEN1, and XPG sgRNA targeting regions are listed in S6 Table.

For indel sequencing analysis of XPF, Mus81, FEN1, and XPG genes, total genomic DNA from the control and knockout cells were extracted using the DNeasy blood and tissue kit (Qiagen, 69506) according to the manufacturer’s protocol. The genomic sequence region spanning each CRISPR sgRNA targeting site was amplified by PCR using the corresponding T7E1 PCR primers (S6 Table), and the PCR amplicon was cloned into pGEM-T Easy vector (Promega, A1360). Approximately 25 plasmid clones were subjected to Sanger sequencing, the major valid sequences of XPF, Mus81, FEN1, and XPG DNA obtained from the control and knockout cells were aligned to analyze the indel mutations.

### Western blot

Western blot was performed as previously described [75] using primary antibodies against, XPF (Invitrogen, MA5-12060), Mus81 (SCBT, sc-47692), FEN1 (TREVIGEN, 4410-PC-100), XPG (Invitrogen, PA5-29168), and β-actin (SCBT, sc-47778). The coding sequences for the epitopes recognized by the selected XPF, Mus81, FEN1, and XPG antibodies do not overlap with the gene-targeting sites of the designed sgRNAs.

### HBV and HDV infection and immunofluorescence

The preparation of cell culture-derived HBV and HDV inocula, virus infection of HepG2-NTCP and PHH cells, intracellular HBcAg and HDδAg immunofluorescence, and extracellular HBeAg chemiluminescent assay (CLIA) were performed as previously described [46,68,76,77].

### Mus81-Eme1 *in vitro* cleavage assay

The *Escherichia coli* Rosetta (DE3) co-transformed by plasmids pET-duet-hEme1 plus pCDF-duet-hMus81 or pCDF-duet-hMus81(T383R+A387R) were cultured in LB medium. His-tagged hMus81-Eme1 complex was purified by a Ni 2+-NTA affinity chromatography as previously described [40]. HBV TR oligoes 1, 2, and 3 (Fig 7 and S7 Table) were synthesized by Genscript. Oligoes 2 and 3 were labeled with IRDye 800 CW maleimide by the 5’ EndTag DNA/RNA Labeling Kit (Vector Labs, MB-9001). The TR DNA substrates were generated as follows: for substrate 1 with 3’ TR-flap, oligo 1 was annealed with unlabeled oligo 3 first in ddH_2_O by heating the mixture at 95°C for 5 min and cooling down to room temperature, then the IRDye-labeled oligo 2 was added to anneal with the 3’ portion of oligo 1, the temperature was raised up to 40°C for 5 min and then moved down to room temperature to complete the reaction. The molar ratio of input oligo 1: oligo 3: labeled oligo 2 is 1: 2: 0.2. Substrate 2 with 5’ TR-flap was prepared similarly, except that oligo 1 was annealed with the unlabeled oligo 2 first and then the labeled oligo 3 (molar ratio 1: 2: 0.2). The assembled DNA substrates were subjected to Mus81-Eme1 cleavage assay in the following reaction mixture [40]: 20 nM fluorescent DNA substrate, purified Mus81-Eme1 at indicated concentrations, 25 mM Tris-HCl (pH 8.0), 5 mM β-mercaptoethanol, 100 μg/ml BSA, 5% Glycerol, 10 mM MgCl_2_, nuclease-free was supplied to bring the reaction volume to 50 μl. The reaction was performed at 37°C for 60 min and then stopped by incubating with reaction stop buffer (0.3% SDS, 5 mM EDTA, pH 8.0, and 0.1 mg/ml proteinase K) for 30 min at 37°C. The reaction products were resolved in 10% native polyacrylamide gel, followed by signal acquisition by LI-COR Odyssey Fc.

### Nuclear DP-rcDNA 5’ and 3’ RACE-NGS and bioinformatic analyses

HepAD38 control, FEN1 K.O., and Mus81 K.O. cells were cultured in the absence of tet for 14 days. The cell fractionations were performed using Qproteome Cell Compartment Kit (QIAGEN, 37502) by following the manufacturer’s directions. The purity of cytoplasmic and nuclear fractions was confirmed by measuring cytoplasm- and nucleus-specific protein markers, specifically GAPDH and Lamin A/C, by Western blot assay. The nuclear Hirt DNA was extracted, and gel purified as previously described [26,39], and dissolved in 20 μl TE buffer, followed by denaturation at 95°C for 10 min and then placed on ice. 1 μl of the denatured nuclear Hirt DNA was mixed with 1 μl 10 × T4 RNA ligase buffer (NEB, B0216), 4 μl 50% PEG-8000 (working concentration is 20%), 1 μl T4 RNA ligase 1 (NEB, M0204), 1 μl 10 mM ATP, 1 μl 20 mM Anchor oligo (S8 Table), 1 μl nuclease-free water was added to make a 10 μl T4 ligation reaction volume. The ligation reaction was kept in room temperature overnight. 3 μl of ligation product were used as template for the Terra Direct PCR reaction (Takara, 639270). Anchor primer and HBV-specific primer sets (S8 Table) were used to amplify the 5’ and 3’ terminal sequences on the (-) strand of nuclear DP-rcDNA. The PCR reaction mixture was denatured at 98°C for 2 min, and the amplification was set for 35 cycles of 98°C for 10 sec, 60°C for 15 sec and 68°C for 30 sec according to the manufacturer’s manual.

The above PCR products (400–450 bp) amplifying junctions of sequence adaptors and either HBV (-) strand DNA 5’ or 3’ end were subjected to DNA sequencing library preparation as follows: 55 μl of the diluted DNA (5 ng/μl) was sheared using Covaris M220 with the following settings: Duty Factor 10%, Power 75W, Cycles/Burst 200, Duration 80 sec at 20°C. The aimed insert size is between 300–350 bp. The fragmented DNA was purified using the MinElute PCR purification kit (Qiagen, 28004) and eluted in 10 μl of Nuclease-free water.

DNA libraries were constructed from the fragmented DNAs using the Tecan Celero DNA-Seq kit (Tecan, 0360A-UDI) with the streamlined workflow starting with end repair of fragmented DNA followed by adaptor ligation and amplification to produce the final library. Unique dual-indexes set A (UDI-A) were introduced to the libraries and the barcoded libraries are compatible with Illumina NGS platforms for multiplex sequencing. Amplified libraries were purified with CleanNGS DNA &RNA SPRI Beads (BullDog Bio, CNGS050) and eluted in 20 μl of DNA Resuspension Buffer (DR1) (provided in the kit). DNA libraries were quantified in molarity using the novel NuQuant library quantification method on a fluorometer (Qubit, Thermo Fisher). DNA libraries were visualized on an Agilent 2100 bioanalyzer using the High Sensitivity DNA Chip (Agilent Technologies, 5067–4626). Libraries were pooled at equimolar concentrations and the library pool was sequenced on Illumina MiSeq for 2×151bp run, and 22K-57K reads per sample were received. To achieve a greater sequencing depth, the 3’ RACE PCR products from the HepAD38 Mus81 K.O. cells were sequenced on NextSeq 2000 using XLEP chemistry for 2×151bp run. The sequencing was performed in the Cancer Genomics Facility of UPMC Hillman Cancer Center.

The sequences of HBV DNA RACE fragments were detected and quantified through motif search function from qualified sequencing reads through licensed server connected to Qiagen CLC Genomics Workbench 24. Raw sequence reads were first mapped to host genome: Homo_sapiens_hg19-2024-02-23-16-55 to remove any host genome-derived reads. Un-mapped paired reads were subjected to motif search using the RACE anchor-HBV chimeric junctions as the Search Strings shown in S9 Table with Accuracy (%) = 100 for each specific HBV RACE ID. The motif search for sample HepAD38 Mus81 K.O. 3’ RACE-NGS was carried out repeatedly from three 100K reads sub-lists, and the number of detected reads was determined from the average of these three repeats. The frequency of each HBV RACE ID was calculated as follows: [HBV 5’ RACE frequency = # of positively detected ID specific reads (from both strands)/ total # of all positive reads from all 5’ RACE events ×100%] and [HBV 3’ RACE frequency = # of positively detected ID specific reads (from both strands)/ total # of all positive reads from all 3’ RACE events ×100%].

### Reverse transcription (RT)-PCR

RT-PCR assay was employed to detect the mRNA of Mus81 and FEN1 in PHH cells. Total RNA was extracted from PHH cells using TRI Reagent (Millipore Sigma, T9424). 1 μg of total RNA was treated with DNase I (Promega, M6101), followed by 70°C inactivation. The DNase-treated RNA samples were then subjected to RT using SuperScript IV VILO Master Mix (ThermoFisher Scientific, 11756050). Terra PCR Direct Polymerase Mix (TAKARA, 639271) was used to amplify the gene targets of Mus81, FEN1, and GAPDH with primers listed in S10 Table. PCR reactions were performed without the prior RT step in parallel to ensure that any RT-PCR amplification observed was not due to residual genomic DNA contamination in the DNase I-treated RNA samples.

### Statistical analysis

Data were analyzed using Prism 10 (GraphPad Software) with two-tailed unpaired t-tests. Data are expressed as mean ± standard deviation (SD), and p-values of <0.05 are considered statistically significant.

## Supporting information

S1 Fig HBV DNA species- and strand-specific PCR of TR region. (A) HBV rcDNA (+) strand-specific PCR amplification of the TR-complementary region. The designed PCR strategy is illustrated. Rrc: Reverse primer of rcDNA; Frc: Forward primer of rcDNA; Ry: Reverse anchor primer. Red lines indicate non-HBV sequences. The two-step amplification starts with Rrc primer extension using (+) strand as template, followed by PCR amplification using (-) strand-targeting primer Frc and primer Ry derived from the non-HBV portion of Rrc, allowing specific amplification of a rcDNA (+) strand region containing sequence complementary to the TR sequence on (-) strand DNA. In contrast, the dslDNA cannot be amplified due to the split PCR target region on both blunt ends. To validate the rcDNA (+) strand-specific PCR, the 458-bp PCR products of HBV cytoplasmic core DNA from induced HepDES19 and HepDES-C1822G cells were verified by agarose gel, and no PCR product was detected when the purified HBV (-) strand ssDNA was used as template. (B) HBV cccDNA-specific PCR amplification of TR region. Total Hirt DNA extracted from induced HepDES19 and HepDES-C1822G cells was heat denatured to convert protein-free rcDNA and dslDNA into ssDNA, followed by PSAD digestion. The remaining PSAD-resistant cccDNA was subjected to PCR amplification using forward and reverse primers (Fccc and Rccc). The 211-bp TR-containing PCR product of cccDNA was confirmed by agarose gel.
(TIF)

S2 Fig CRISPR/Cas9-mediated XPF knockout in HepAD38 cells. (A) Schematic illustration of XPF gene locus. The green boxes indicate exons, and the solid lines indicate introns. The designed sgRNA is shown in nucleotide sequence (green) with adjacent PAM sequence (red), and its corresponding targeting site in XPF gene is marked by an arrow. (B) XPF gene indel sequencing results. Sequence alignment of the XPF sgRNA-targeting region from HepAD38 control and XPF K.O. cells revealed deletion (clone c5) and insertion (clone c13) causing frameshift and premature termination of XPF ORF in the knockout cells. Nucleotide insertions are in bold red, and deletions are indicated with dashes. Stop codon is indicated with asterisk. The deduced amino acids are shown in one-letter codes underneath the DNA sequence.
(TIF)

S3 Fig Validation of a method for improving cccDNA detection by Southern blot. (A) Schematic diagram of HBV Hirt DNA treatment. HBV Hirt DNA contains protein-free rcDNA, dslDNA, and cccDNA. 85°C heat denaturation transforms rcDNA and dslDNA into ssDNA, while cccDNA remains intact. A subsequent EcoRI digestion linearizes cccDNA into dlsDNA. (B) Method Validation. HepAD38 cells were induced in tet-free medium for 6 and 12 days and subjected to cytoplasmic HBV core DNA and total Hirt DNA extractions, respectively. The DNA samples were left untreated or treated by heat denaturation or heat denaturation plus EcoRI digestion, followed by Southern blot analysis.
(TIF)

S4 Fig CRISPR/Cas9-mediated Mus81 knockout in HepAD38 cells. (A) Schematic illustration of Mus81 gene locus. The green boxes indicate exons, and the solid lines indicate introns. The designed sgRNA is shown in nucleotide sequence (green) with adjacent PAM sequence (red), and its corresponding targeting site in Mus81 gene is marked by an arrow. (B) Mus81 gene indel sequencing results. Sequence alignment of the Mus81 sgRNA-targeting region from HepAD38 control and Mus81 K.O. cells revealed mutations causing frameshift and premature termination (clone c1) or in-frame deletions (clone c7 and c8) in the knockout cells.
(TIF)

S5 Fig CRISPR/Cas9-mediated Mus81 knockout in HepG2-NTCP cells. (A) Mus81 knockout was performed in HepG2-NTCP cells using CRISPR/Cas9 with the sgRNA described in S4A Fig. Five Mus81 K.O. clones (c6, c7, c8, c10 and c11) were obtained and subjected to T7E1 assay and Western blot. (B) Indel sequencing was conducted with HepG2-NTCP control K.O. cells and Mus81 K.O. cells (clone c7). Sequence alignment of the sgRNA-targeting region in Mus81 gene loci revealed a single nucleotide deletion causing frameshift and premature termination of Mus81 ORF in clone c7.
(TIF)

S6 Fig Mus81 knockout has no influence on HDV infection. HepG2-NTCP control and Mus81 K.O. cells were infected with HDV at MOI 200 and 500 for 5 days, followed by HDδAg immunofluorescence assay.
(TIF)

S7 Fig CRISPR/Cas9-mediated FEN1 knockout in HepAD38 cells. (A) Schematic illustration of FEN1 gene locus. The green boxes indicate exons, and the solid lines indicate introns. The designed sgRNA is shown in nucleotide sequence (green) with adjacent PAM sequence (red), and its corresponding targeting site in FEN1 gene is marked. (B) FEN1 gene indel sequencing results. Sequence alignment of the sgRNA-targeting region in FEN1 gene loci from HepAD38 control and FEN1 K.O. cells revealed a single nucleotide deletion causing frameshift and premature termination of FEN1 ORF in HepAD38 FEN1 K.O. cells.
(TIF)

S8 Fig CRISPR/Cas9-mediated XPG knockout in HepAD38 cells. (A) Schematic illustration of XPG gene locus. The green boxes indicate exons, and the solid lines indicate introns. The designed sgRNA is shown in nucleotide sequence (green) with adjacent PAM sequence (red), and its corresponding targeting site in XPG gene is marked. (B) XPG gene indel sequencing results. Sequence alignment of the sgRNA-targeting region in XPG gene loci from HepAD38 control and XPG K.O. cells revealed a single nucleotide insertion causing frameshift and premature termination of XPG ORF in HepAD38 XPG K.O. cells.
(TIF)

S1 Table Summary of nuclear HBV DP-rcDNA (-) strand 5’ and 3’ RACE-NGS analyses. (PDF)

S2 Table Oligos for cytoplasmic HBV rcDNA (-) strand 3’ RACE. (PDF)

S3 Table Oligos for HBV rcDNA TR-complementary (+) strand PCR amplification. (PDF)

S4 Table Oligos for HBV cccDNA TR region amplification. (PDF)

S5 Table Oligo pairs of CRISPR sgRNA. (PDF)

S6 Table PCR primer pairs for T7E1 assay. (PDF)

S7 Table HBV oligos for generating 5’ and 3’ TR-flap structures. (PDF)

S8 Table Oligos for nuclear HBV DP-rcDNA (-) strand 5’ and 3’ RACE. (PDF)

S9 Table Search strings for nuclear HBV DP-rcDNA (-) strand 5’ and 3’ RACE-NGS. (PDF)

S10 Table RT-PCR primers. (PDF)
