# Supplementary figures and images for: Host 3’ flap endonuclease Mus81 plays a critical role in trimming the terminal redundancy of hepatitis B virus relaxed circular DNA during covalently closed circular DNA formation
Source: PLoS Pathog. 2025 Feb 6;21(2):e1012918. doi: 10.1371/journal.ppat.1012918 (PMC11801639; doi:10.1371/journal.ppat.1012918)

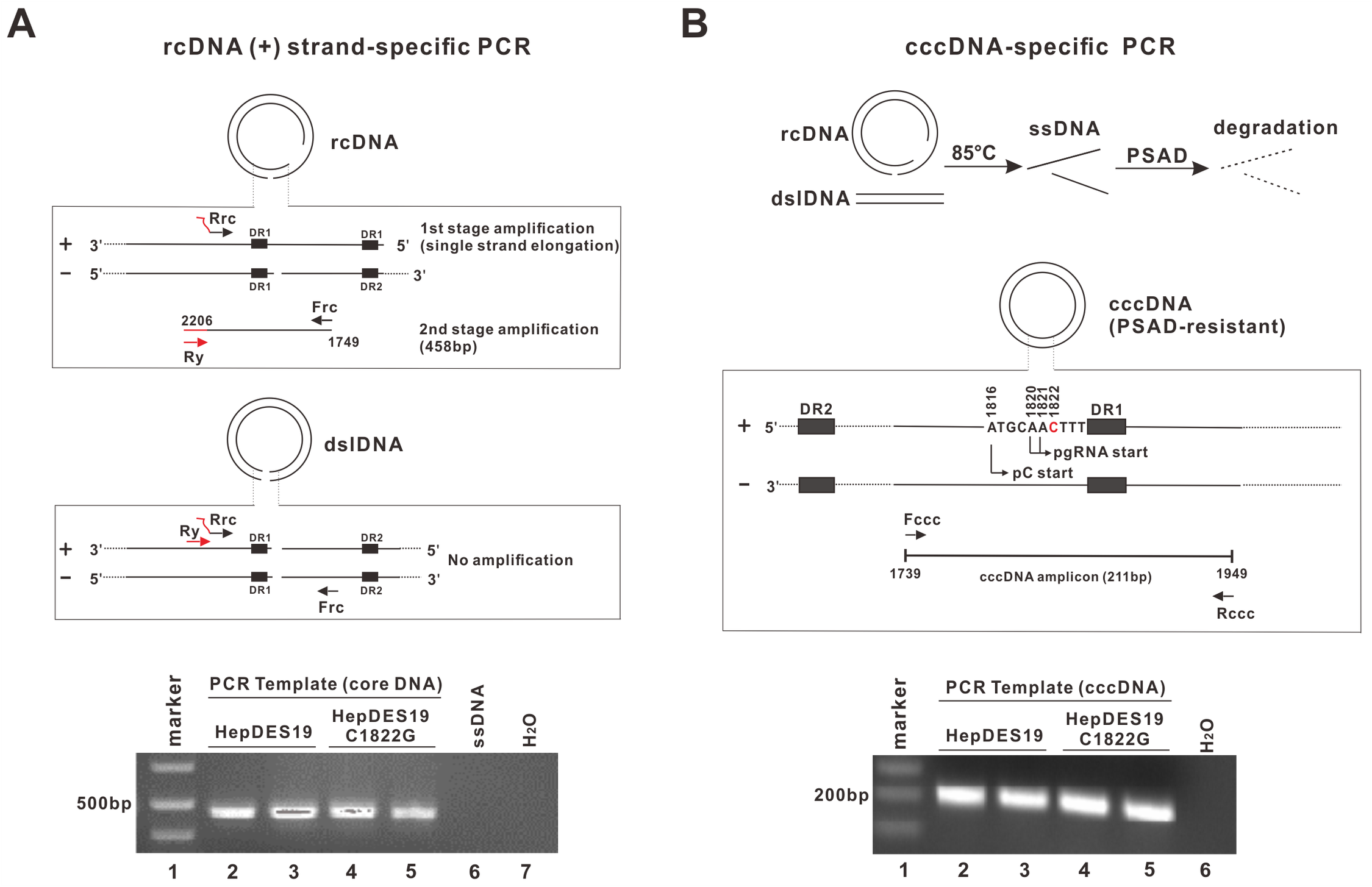

Supplement: S1 Fig — (A) HBV rcDNA (+) strand-specific PCR amplification of the TR-complementary region. The designed PCR strategy is illustrated. Rrc: Reverse primer of rcDNA; Frc: Forward primer of rcDNA; Ry: Reverse anchor primer. Red lines indicate non-HBV sequences. The two-step amplification starts with Rrc primer extension using (+) strand as template, followed by PCR amplification using (-) strand-targeting primer Frc and primer Ry derived from the non-HBV portion of Rrc, allowing specific amplification of a rcDNA (+) strand region containing sequence complementary to the TR sequence on (-) strand DNA. In contrast, the dslDNA cannot be amplified due to the split PCR target region on both blunt ends. To validate the rcDNA (+) strand-specific PCR, the 458-bp PCR products of HBV cytoplasmic core DNA from induced HepDES19 and HepDES-C1822G cells were verified by agarose gel, and no PCR product was detected when the purified HBV (-) strand ssDNA was used as template. (B) HBV cccDNA-specific PCR amplification of TR region. Total Hirt DNA extracted from induced HepDES19 and HepDES-C1822G cells was heat denatured to convert protein-free rcDNA and dslDNA into ssDNA, followed by PSAD digestion. The remaining PSAD-resistant cccDNA was subjected to PCR amplification using forward and reverse primers (Fccc and Rccc). The 211-bp TR-containing PCR product of cccDNA was confirmed by agarose gel. (TIF) [file ppat.1012918.s001.tif]

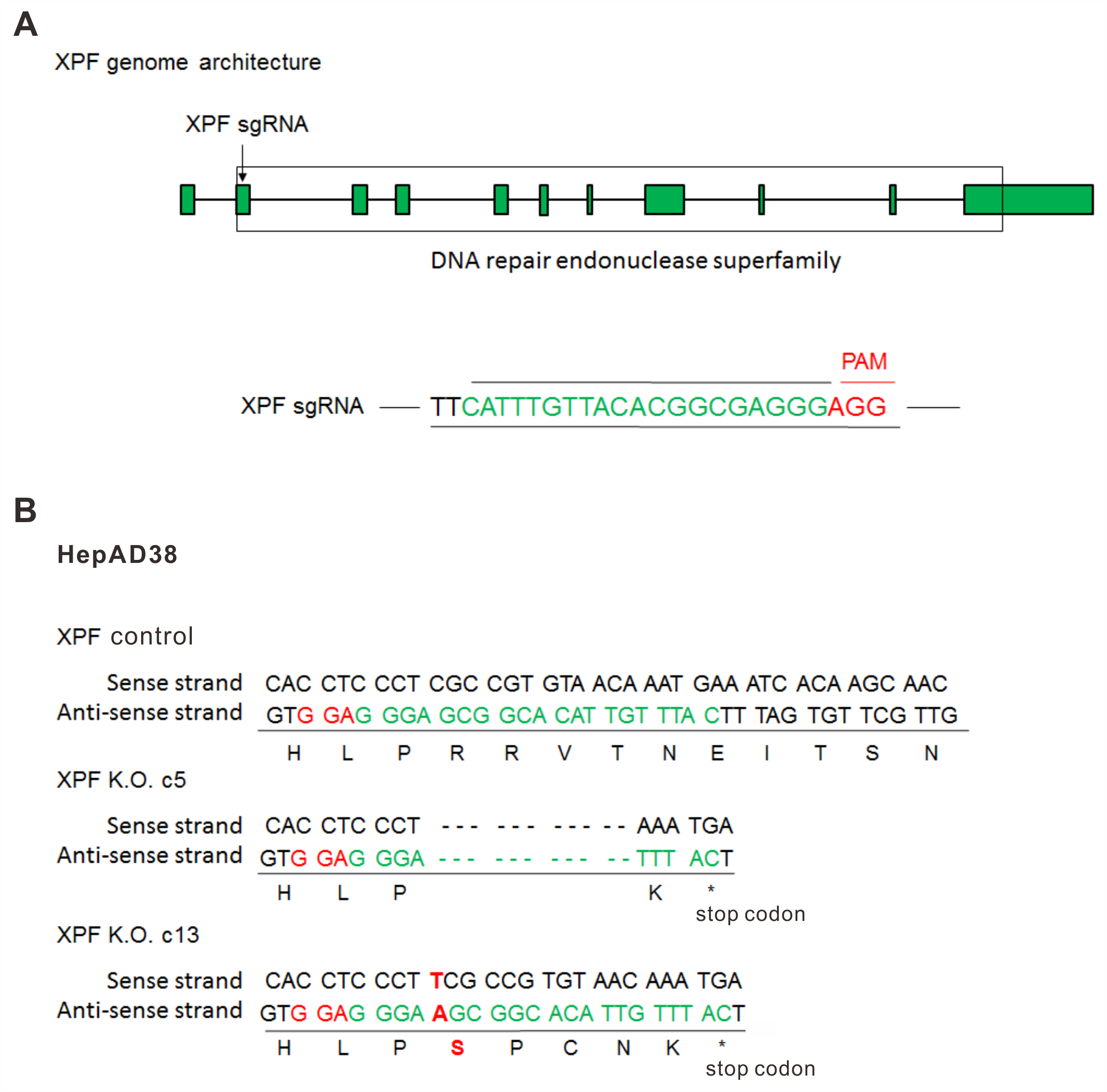

Supplement: S2 Fig — (A) Schematic illustration of XPF gene locus. The green boxes indicate exons, and the solid lines indicate introns. The designed sgRNA is shown in nucleotide sequence (green) with adjacent PAM sequence (red), and its corresponding targeting site in XPF gene is marked by an arrow. (B) XPF gene indel sequencing results. Sequence alignment of the XPF sgRNA-targeting region from HepAD38 control and XPF K.O. cells revealed deletion (clone c5) and insertion (clone c13) causing frameshift and premature termination of XPF ORF in the knockout cells. Nucleotide insertions are in bold red, and deletions are indicated with dashes. Stop codon is indicated with asterisk. The deduced amino acids are shown in one-letter codes underneath the DNA sequence. (TIF) [file ppat.1012918.s002.tif]

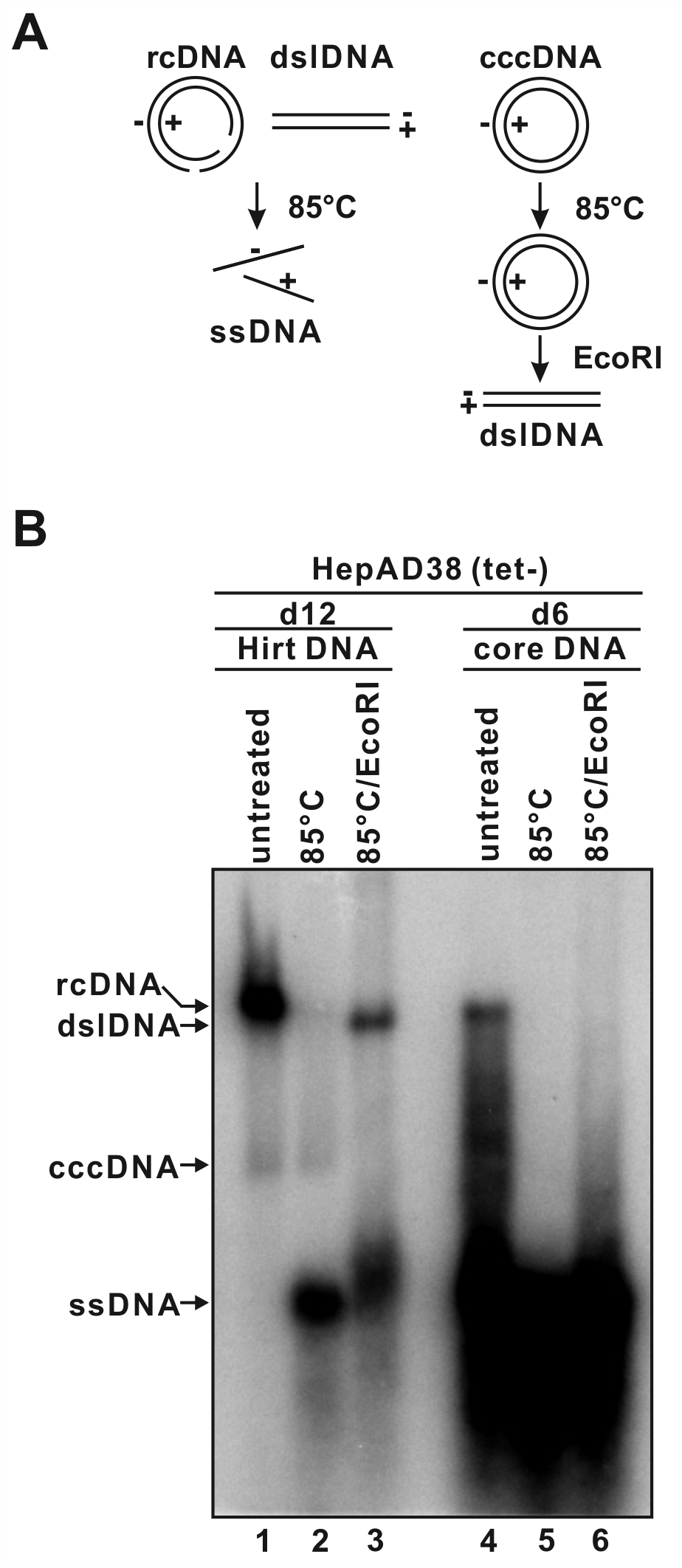

Supplement: S3 Fig — (A) Schematic diagram of HBV Hirt DNA treatment. HBV Hirt DNA contains protein-free rcDNA, dslDNA, and cccDNA. 85°C heat denaturation transforms rcDNA and dslDNA into ssDNA, while cccDNA remains intact. A subsequent EcoRI digestion linearizes cccDNA into dlsDNA. (B) Method Validation. HepAD38 cells were induced in tet-free medium for 6 and 12 days and subjected to cytoplasmic HBV core DNA and total Hirt DNA extractions, respectively. The DNA samples were left untreated or treated by heat denaturation or heat denaturation plus EcoRI digestion, followed by Southern blot analysis. (TIF) [file ppat.1012918.s003.tif]

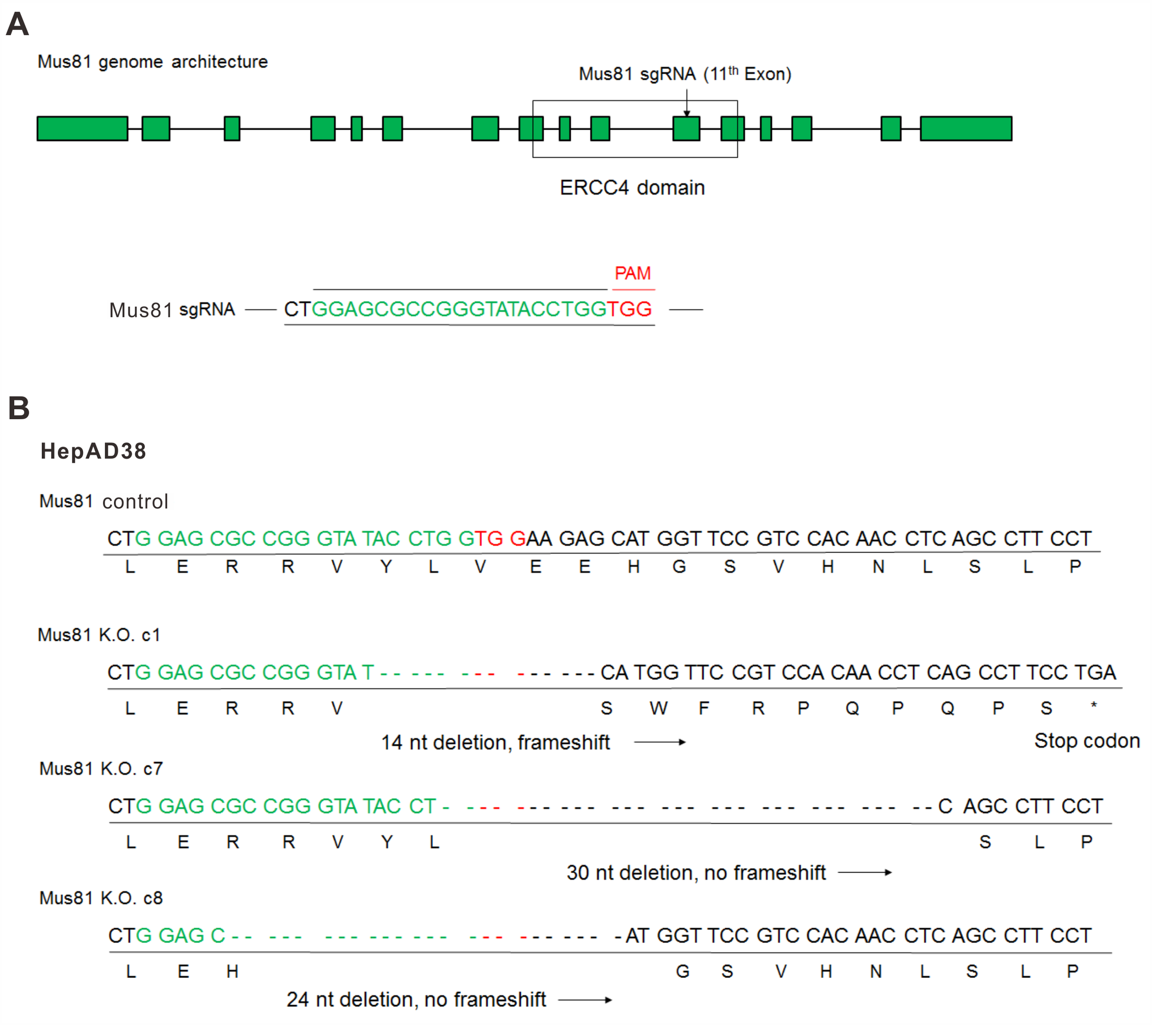

Supplement: S4 Fig — (A) Schematic illustration of Mus81 gene locus. The green boxes indicate exons, and the solid lines indicate introns. The designed sgRNA is shown in nucleotide sequence (green) with adjacent PAM sequence (red), and its corresponding targeting site in Mus81 gene is marked by an arrow. (B) Mus81 gene indel sequencing results. Sequence alignment of the Mus81 sgRNA-targeting region from HepAD38 control and Mus81 K.O. cells revealed mutations causing frameshift and premature termination (clone c1) or in-frame deletions (clone c7 and c8) in the knockout cells. (TIF) [file ppat.1012918.s004.tif]

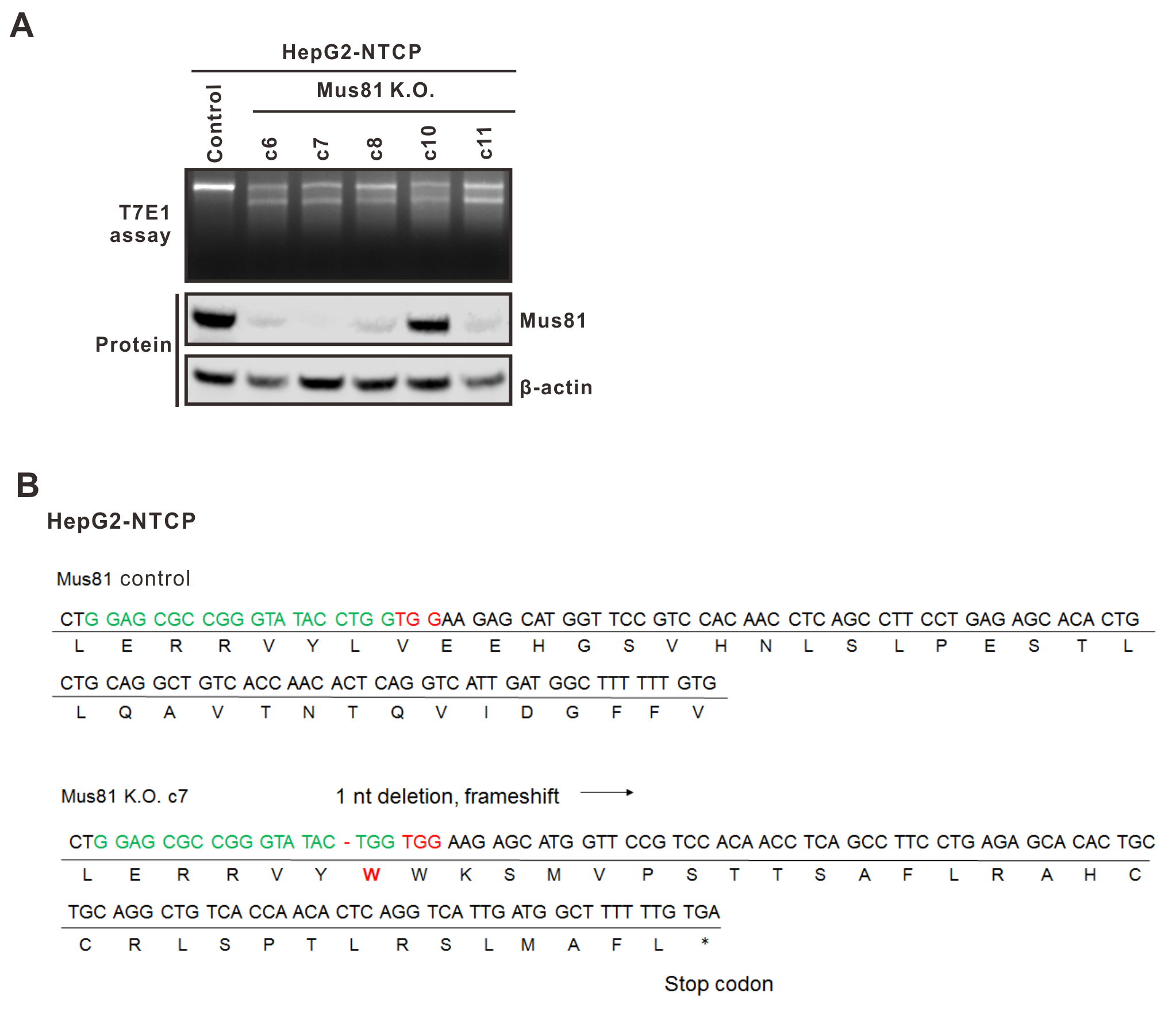

Supplement: S5 Fig — (A) Mus81 knockout was performed in HepG2-NTCP cells using CRISPR/Cas9 with the sgRNA described in S4A Fig. Five Mus81 K.O. clones (c6, c7, c8, c10 and c11) were obtained and subjected to T7E1 assay and Western blot. (B) Indel sequencing was conducted with HepG2-NTCP control K.O. cells and Mus81 K.O. cells (clone c7). Sequence alignment of the sgRNA-targeting region in Mus81 gene loci revealed a single nucleotide deletion causing frameshift and premature termination of Mus81 ORF in clone c7. (TIF) [file ppat.1012918.s005.tif]

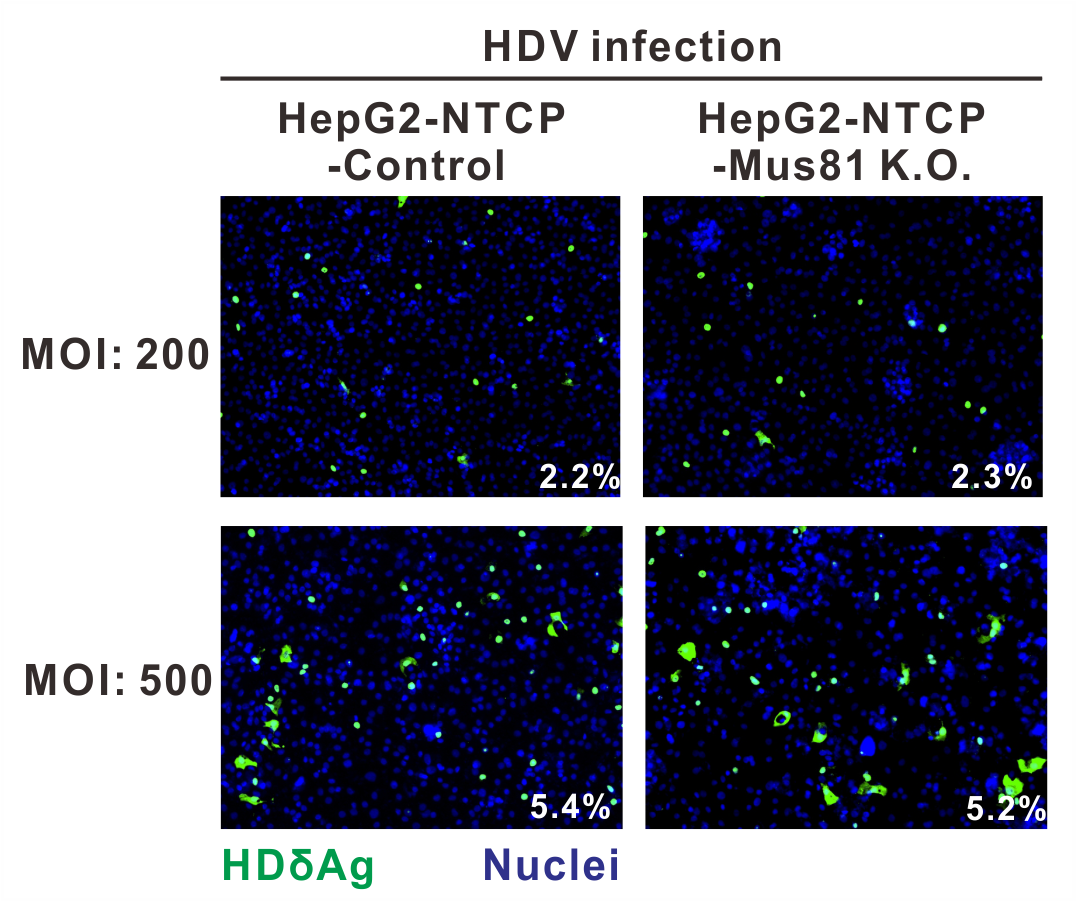

Supplement: S6 Fig — HepG2-NTCP control and Mus81 K.O. cells were infected with HDV at MOI 200 and 500 for 5 days, followed by HDδAg immunofluorescence assay. (TIF) [file ppat.1012918.s006.tif]

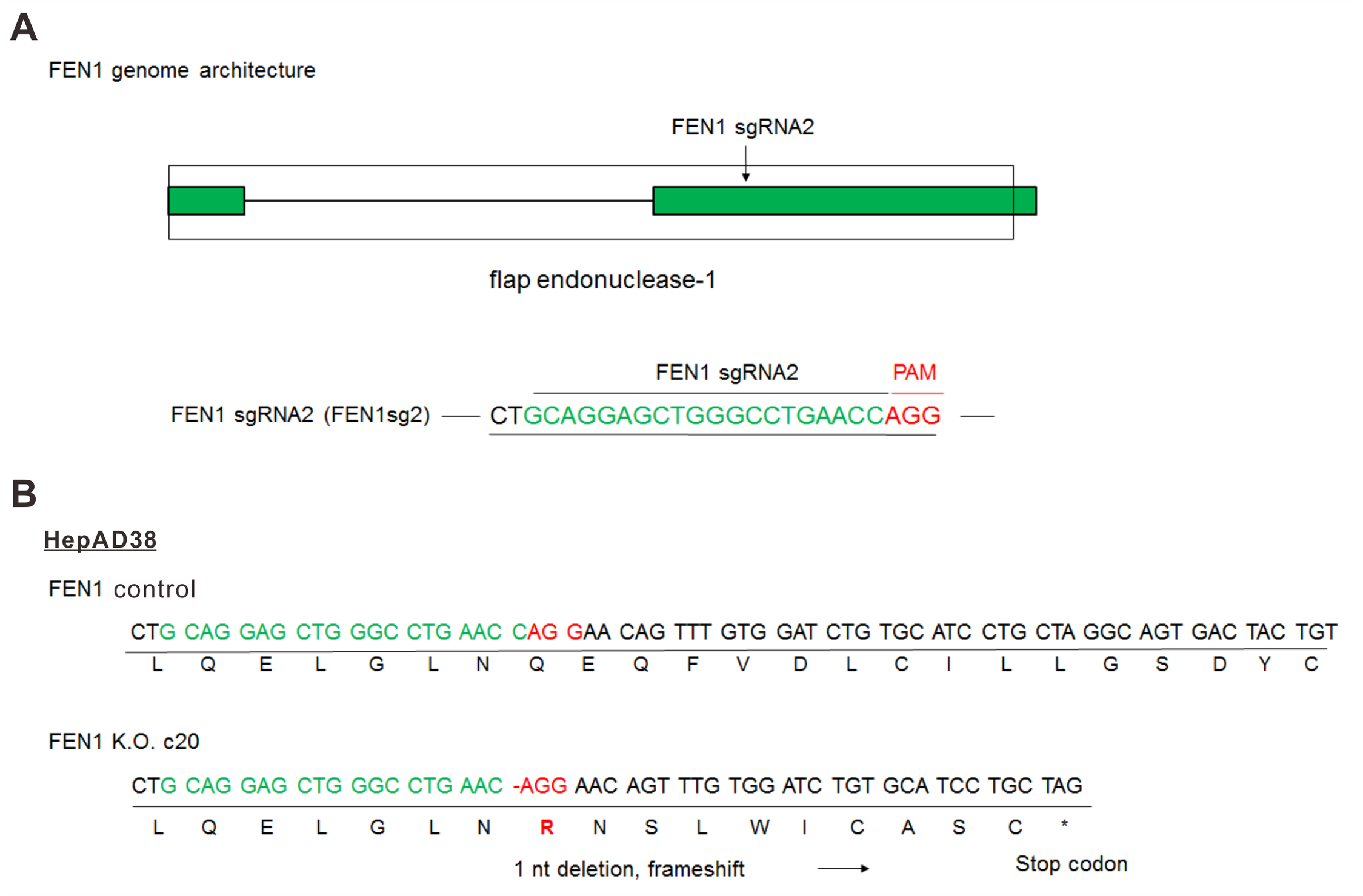

Supplement: S7 Fig — (A) Schematic illustration of FEN1 gene locus. The green boxes indicate exons, and the solid lines indicate introns. The designed sgRNA is shown in nucleotide sequence (green) with adjacent PAM sequence (red), and its corresponding targeting site in FEN1 gene is marked. (B) FEN1 gene indel sequencing results. Sequence alignment of the sgRNA-targeting region in FEN1 gene loci from HepAD38 control and FEN1 K.O. cells revealed a single nucleotide deletion causing frameshift and premature termination of FEN1 ORF in HepAD38 FEN1 K.O. cells. (TIF) [file ppat.1012918.s007.tif]

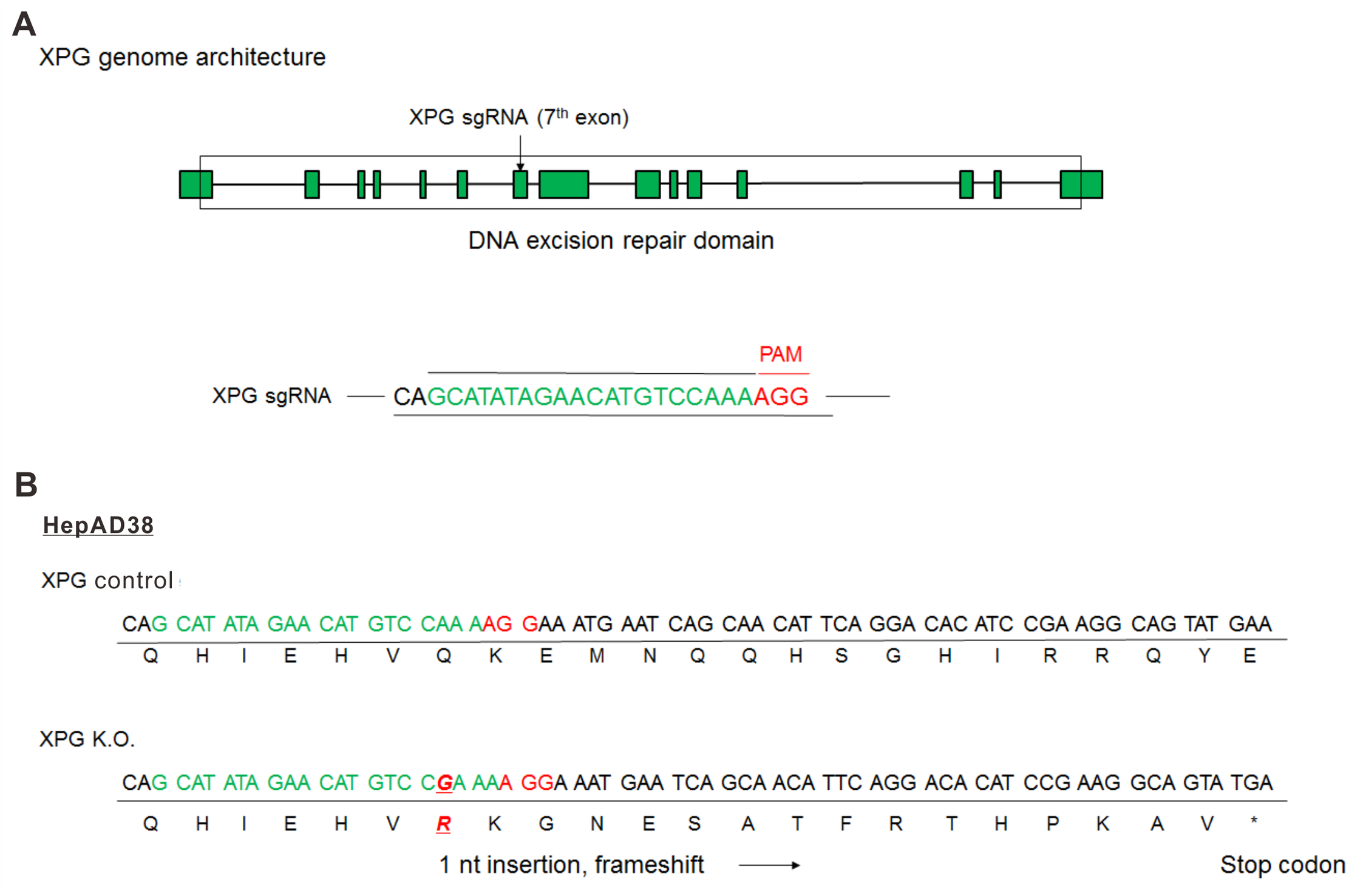

Supplement: S8 Fig — (A) Schematic illustration of XPG gene locus. The green boxes indicate exons, and the solid lines indicate introns. The designed sgRNA is shown in nucleotide sequence (green) with adjacent PAM sequence (red), and its corresponding targeting site in XPG gene is marked. (B) XPG gene indel sequencing results. Sequence alignment of the sgRNA-targeting region in XPG gene loci from HepAD38 control and XPG K.O. cells revealed a single nucleotide insertion causing frameshift and premature termination of XPG ORF in HepAD38 XPG K.O. cells. (TIF) [file ppat.1012918.s008.tif]
