# Supplementary material for: Host 3’ flap endonuclease Mus81 plays a critical role in trimming the terminal redundancy of hepatitis B virus relaxed circular DNA during covalently closed circular DNA formation
Source: PLoS Pathog. 2025 Feb 6;21(2):e1012918. doi: 10.1371/journal.ppat.1012918 (PMC11801639; doi:10.1371/journal.ppat.1012918)
Supplement: S1 Table — (PDF) [file ppat.1012918.s009.pdf]

S1 Table. Summary of nuclear HBV DP-rcDNA (-) strand 5' and 3' RACE-NGS analyses.

	**HepAD38**					
	**Control**		**FEN1 K.O.**		**Mus81 K.O.**	
**5' end**	**Counts** (total: 1657)	**Frequency**	**Counts** (total: 1012)	**frequency**	**Counts** (total: 1499)	**frequency**
1829T	8	0.5%	11	1.1%	23	1.5%
1828G	423	25.5%	456	45.1%	452	30.2%
1827A	55	3.3%	50	4.9%	128	8.5%
1826A	48	2.9%	32	3.2%	45	3.0%
1825A	177	10.7%	67	6.6%	180	12.0%
1824A	167	10.1%	105	10.4%	182	12.1%
1823A	75	4.5%	66	6.5%	67	4.5%
1822G	51	3.1%	56	5.5%	49	3.3%
1821T	330	19.9%	77	7.6%	189	12.6%
1820T	203	12.3%	11	1.1%	113	7.5%
1819G	56	3.4%	60	5.9%	37	2.5%
1818C	57	3.4%	17	1.7%	27	1.8%
1817A	7	0.4%	4	0.4%	7	0.5%
**3' end**	**Counts** (total: 993)	**Frequency**	**Counts** (total: 359)	**frequency**	**Counts** (total: 3487)	**frequency**
1820T	43	4.33%	16	4.5%	109	3.1%
1821T	212	21.35%	20	5.6%	940	27.0%
1822G	266	26.79%	122	34.0%	462	13.2%
1823A	99	9.97%	71	19.8%	744	21.3%
1824A	56	5.64%	25	7.0%	575	16.5%
1825A	63	6.34%	13	3.6%	433	12.4%
1826A	169	17.02%	31	8.6%	106	3.0%
1827A	41	4.13%	24	6.7%	63	1.8%
1828G	15	1.51%	11	3.1%	25	0.7%
1829T	13	1.31%	11	3.1%	8	0.2%
1830G	8	0.81%	6	1.7%	11	0.3%
1831G	8	0.81%	9	2.5%	12	0.4%
