# Supplementary material for: Host 3’ flap endonuclease Mus81 plays a critical role in trimming the terminal redundancy of hepatitis B virus relaxed circular DNA during covalently closed circular DNA formation
Source: PLoS Pathog. 2025 Feb 6;21(2):e1012918. doi: 10.1371/journal.ppat.1012918 (PMC11801639; doi:10.1371/journal.ppat.1012918)
Supplement: S2 Table — (PDF) [file ppat.1012918.s010.pdf]

S2 Table. Oligos for cytoplasmic HBV rcDNA (-) strand 3' RACE

**Oligo**	**Sequence (5'→3' orientation)**
Anchor	PO_4-AGGTACTCTATCCTAGACCGTCACCATTTGCTACATGCTG ACAGCCTA-PO_4
HBV Primer	AAGGCCTTCACAACTATCCTATCCCCGTA (nt 2337-2309)
Anchor primer	AGCAAATGGTGACGGTCTAGGATAGAGTAC
