# Supplementary material for: Host 3’ flap endonuclease Mus81 plays a critical role in trimming the terminal redundancy of hepatitis B virus relaxed circular DNA during covalently closed circular DNA formation
Source: PLoS Pathog. 2025 Feb 6;21(2):e1012918. doi: 10.1371/journal.ppat.1012918 (PMC11801639; doi:10.1371/journal.ppat.1012918)
Supplement: S3 Table — (PDF) [file ppat.1012918.s011.pdf]

S3 Table. Oligos for HBV rcDNA TR-complementary (+) strand PCR amplification.

Oligo	Sequence (5’→3’ orientation)
Rrc	ATCTATGTGCATCCGACAAGCACAAGAGTTGCCTGAACTTTAGGC (nt 2206-2183)
Frc1	GGTGAGCAAGCAGGAGATTAGATTAAAGGTCT (nt 1749-1769)
Frc2	AGTTCATACGGCAGGAGATTAGATTAAAGGTCT (nt 1749-1769)
Ry	ATCTATGTGCATCCGACAAGC

Note: Underlined nt represented non-HBV sequence. The first 11 bases of Frc1 and Frc2 are barcodes for NGS.
