# Supplementary material for: Host 3’ flap endonuclease Mus81 plays a critical role in trimming the terminal redundancy of hepatitis B virus relaxed circular DNA during covalently closed circular DNA formation
Source: PLoS Pathog. 2025 Feb 6;21(2):e1012918. doi: 10.1371/journal.ppat.1012918 (PMC11801639; doi:10.1371/journal.ppat.1012918)
Supplement: S4 Table — (PDF) [file ppat.1012918.s012.pdf]

S4 Table. Oligos for HBV cccDNA TR region amplification.

Oligo	Sequence (5'→3' orientation)
Fccc	GAGTTGGGGGAGGAGATTAGA（nt 1739-1759）
Rccc	AGTAACTCCACAGTAGCTCCAAATT (nt 1949-1925)
