# Supplementary material for: Host 3’ flap endonuclease Mus81 plays a critical role in trimming the terminal redundancy of hepatitis B virus relaxed circular DNA during covalently closed circular DNA formation
Source: PLoS Pathog. 2025 Feb 6;21(2):e1012918. doi: 10.1371/journal.ppat.1012918 (PMC11801639; doi:10.1371/journal.ppat.1012918)
Supplement: S5 Table — (PDF) [file ppat.1012918.s013.pdf]

S5 Table. Oligo pairs of CRISPR sgRNA.

Oligo	Sequence (5’→3’ orientation)
XPF sgRNA forward (F)	caccgCATTTGTTACACGGCGAGGG
XPF sgRNA reverse (R)	aaacCCCTCGCCGTGTAACAAATGc
Mus81 sgRNA forward (F)	caccgGGAGCGCCGGGTATACCTGG
Mus81 sgRNA reverse (R)	aaacCCAGGTATACCCGGCGCTCCc
FEN1 sgRNA forward (F)	caccgGCAGGAGCTGGGCCTGAACC
FEN1 sgRNA reverse (R)	aaacGGTTCAGGCCCAGCTCCTGCc
XPG sgRNA forward (F)	caccgCATATAGAACATGTCCAAA
XPG sgRNA reverse (R)	aaacTTTGGACATGTTCTATATGc

Note: the lowercase letters in the oligo sequences indicate the sticky ends of BsmBI restriction site.
