# Supplementary material for: Host 3’ flap endonuclease Mus81 plays a critical role in trimming the terminal redundancy of hepatitis B virus relaxed circular DNA during covalently closed circular DNA formation
Source: PLoS Pathog. 2025 Feb 6;21(2):e1012918. doi: 10.1371/journal.ppat.1012918 (PMC11801639; doi:10.1371/journal.ppat.1012918)
Supplement: S6 Table — (PDF) [file ppat.1012918.s014.pdf]

S6 Table. PCR primer pairs for T7E1 assay.

Oligo	Sequence (5'→3' orientation)
**HepAD38 cells**	
XPF sgRNA T7E1 forward (F)	ACTGCCCTGTATTAAATAGCCTA
XPF sgRNA T7E1 reverse (R)	AAGCCATTAAGAAATTCGTCA
Mus81 sgRNA T7E1 long forward (F)	GTGGACATTGGCGAGAC
Mus81 sgRNA T7E1 long reverse (R)	CCCGACACTGGGAAAGG
FEN1 sgRNA T7E1 forward (F)	GTATGTCTTTGATGGCAAGCCG
FEN1 sgRNA T7E1 reverse (R)	TGGCACAGGGTACTTGTTGG
XPG sgRNA T7E1 forward (F)	TTGTTTATTTTGCCTTTAGGAG
XPG sgRNA T7E1 reverse (R)	CAGTGTGACAATACGCTTC
**HepG2-NTCP cells**	
Mus81 sgRNA T7E1 short forward (F)	GGCTAACTGGTGGGAACA
Mus81 sgRNA T7E1 short reverse (R)	CTGAGGGAAGAAGGGACTG
