# Supplementary material for: Host 3’ flap endonuclease Mus81 plays a critical role in trimming the terminal redundancy of hepatitis B virus relaxed circular DNA during covalently closed circular DNA formation
Source: PLoS Pathog. 2025 Feb 6;21(2):e1012918. doi: 10.1371/journal.ppat.1012918 (PMC11801639; doi:10.1371/journal.ppat.1012918)
Supplement: S7 Table — (PDF) [file ppat.1012918.s015.pdf]

S7 Table. HBV oligos for generating 5' and 3' TR-flap structures.

Oligo	Sequence (5'→3' orientation)
Oligo 1	CCAGCACCATGCAACTTTTTCACCTCTGCCTAAT (+ strand, nt 1808-1841)
Oligo 2	ATTAGGCAGAGGTGAAAAAGT (- strand, nt 1841-1821)
Oligo 3	GAAAAAGTTGCATGGTGCTGG (- strand, nt 1828-1808)

Note: the underlined nucleotides indicate the TR sequence.
