# Supplementary material for: Host 3’ flap endonuclease Mus81 plays a critical role in trimming the terminal redundancy of hepatitis B virus relaxed circular DNA during covalently closed circular DNA formation
Source: PLoS Pathog. 2025 Feb 6;21(2):e1012918. doi: 10.1371/journal.ppat.1012918 (PMC11801639; doi:10.1371/journal.ppat.1012918)
Supplement: S8 Table — (PDF) [file ppat.1012918.s016.pdf]

S8 Table. Oligos for nuclear HBV DP-rcDNA (-) strand 5’ and 3’ RACE.

Oligo	Sequence (5’→3’ orientation)
**5’ RACE**	
Anchor	OH-AGGTACTCTATCCTAGACCGTCACCATTTGCTACATGCTGAC AGCCTA-OH
HBV Primer	TTTGTTTACGTCCCGTCGGCGCTGAATC (nt 1422-1449)
Anchor primer	ACTCTATCCTAGACCGTCACCATTTGCTAC
**3’ RACE**	
Anchor	PO_4-AGGTACTCTATCCTAGACCGTCACCATTTGCTACATGCTGAC AGCCTA-PO_4
HBV Primer	AAACCACAAGAGTTGCCTGA (nt 2211-2192)
Anchor primer	AGCAAATGGTGACGGTCTAGGATAGAGTAC
