# Supplementary material for: Host 3’ flap endonuclease Mus81 plays a critical role in trimming the terminal redundancy of hepatitis B virus relaxed circular DNA during covalently closed circular DNA formation
Source: PLoS Pathog. 2025 Feb 6;21(2):e1012918. doi: 10.1371/journal.ppat.1012918 (PMC11801639; doi:10.1371/journal.ppat.1012918)
Supplement: S9 Table — (PDF) [file ppat.1012918.s017.pdf]

S9 Table. Search strings for nuclear HBV DP-rcDNA (-) strand 5’ and 3’ RACE-NGS.

**ID**	**Description**	**Search String[1]**
**HBV DP-rcDNA (-) strand 5’ RACE-NGS**		
1829T	Intact 5' end with nt 1829T	**TA**TGAAAAAG
1828G	Intact 5' end with nt 1828G	**TA**GAAAAAG
1827A	Cleave G	**TA**AAAAAGT
1826A	Cleave GA	**TA**AAAAGTT
1825A	Cleave GAA	**TA**AAAGTTG
1824A	Cleave GAAA	**TA**AAGTTGC
1823A	Cleave GAAAA	**TA**AGTTGCA
1822G	Cleave GAAAAA	**TA**GTTGCAT
1821T	Cleave GAAAAAG	**TA**TTGCATG
1820T	Cleave GAAAAAGT	**TA**TGCATGG
1819G	Cleave GAAAAAGTT	**TA**GCATGGT
1818C	Cleave GAAAAAGTTG	**TA**CATGGTG
1817A	Cleave GAAAAAGTTGC	**TA**ATGGTGC
**HBV DP-rcDNA (-) strand 3’ RACE-NGS**		
1820T	Intact 3' end with nt 1820T	AAAGTT**AGG**
1821T	Intact 3' end with nt 1821T	AAAAGT**AGG**
1822G	Cleave T	AAAAAG**AGG**
1823A	Cleave GT	GAAAAA**AGG**
1824A	Cleave AGT	TGAAAA**AGG**
1825A	Cleave AAGT	GTGAAA**AGG**
1826A	Cleave AAAGT	GGTGAA**AGG**
1827A	Cleave AAAAGT	AGGTGA**AGG**
1828G	Cleave AAAAAGT	GAGGTG**AGG**
1829T	Cleave GAAAAAGT	AGAGGT**AGG**
1830G	Cleave TGAAAAAGT	CAGAGG**AGG**
1831G	Cleave GTGAAAAAGT	GCAGAG**AGG**

[1] nucleotide in bold: RACE anchor sequence; underlined: HBV sequence
