# Supplementary material for: Host 3’ flap endonuclease Mus81 plays a critical role in trimming the terminal redundancy of hepatitis B virus relaxed circular DNA during covalently closed circular DNA formation
Source: PLoS Pathog. 2025 Feb 6;21(2):e1012918. doi: 10.1371/journal.ppat.1012918 (PMC11801639; doi:10.1371/journal.ppat.1012918)
Supplement: S10 Table — (PDF) [file ppat.1012918.s018.pdf]

S10 Table. RT-PCR primers.

Oligo	Sequence (5’→3’ orientation)
RT primer	oligo $(dT)_{18}$
Mus81 Fr	CTAGCCAGCCTTTTAACAACA
Mus81 Rv	AGGCATCTTCTACATGACACA
FEN1 Fr	CCGTCTTGTACCCTTAAGAGC
FEN1 Rv	GAAAAGTACCTCTGTGGCCTT
GAPDH Fr	TCTGACTTCAACAGCGACACC
GAPDH Rv	CTGTTGCTGTAGCCAAATTCGTT
